# Discovering 3D hidden elasticity in isotropic and transversely isotropic materials with physics-informed UNets

**DOI:** 10.1016/j.actbio.2024.06.038

**Published:** 2024-07-02

**Authors:** Ali Kamali, Kaveh Laksari

**Affiliations:** a Department of Biomedical Engineering, University of Arizona College of Engineering, Tucson, AZ, USA; b Department of Aerospace and Mechanical Engineering, University of Arizona College of Engineering, Tucson, AZ, USA; c Department of Mechanical Engineering, University of California Riverside, Riverside, CA, USA

**Keywords:** Physics-informed deep learning, Tissue biomechanics, Model-based elastography, Digital volume correlation, Scientific computing

## Abstract

Three-dimensional variation in structural components or fiber alignments results in complex mechanical property distribution in tissues and biomaterials. In this paper, we use a physics-informed UNet-based neural network model (El-UNet) to discover the three-dimensional (3D) internal composition and space-dependent material properties of heterogeneous isotropic and transversely isotropic materials without *a priori* knowledge of the composition. We then show the capabilities of El-UNet by validating against data obtained from finite-element simulations of two soft tissues, namely, brain tissue and articular cartilage, under various loading conditions. We first simulated compressive loading of 3D brain tissue comprising of distinct white matter and gray matter mechanical properties undergoing small strains with isotropic linear elastic behavior, where El-UNet reached mean absolute relative errors under 1.5 % for elastic modulus and Poisson’s ratio estimations across the 3D volume. We showed that the 3D solution achieved by El-UNet was superior to relative stiffness mapping by inverse of axial strain and two-dimensional plane stress/plane strain approximations. Additionally, we simulated a transversely isotropic articular cartilage with known fiber orientations undergoing compressive loading, and accurately estimated the spatial distribution of all five material parameters, with mean absolute relative errors under 5 %. Our work demonstrates the application of the computationally efficient physics-informed El-UNet in 3D elasticity imaging and provides methods for translation to experimental 3D characterization of soft tissues and other materials. The proposed El-UNet offers a powerful tool for both *in vitro* and *ex vivo* tissue analysis, with potential extensions to *in vivo* diagnostics.

## 1. Introduction

The three-dimensional kinematic behavior of materials under loading is an area of interest in various fields such as biomechanics, experimental mechanics, biomedical engineering and materials science [1]. Digital volume correlation (DVC), an extension of the widely known two-dimensional digital image correlation (DIC), is a technique that estimates displacement and strain distributions in three-dimensional (3D) materials and tissues under deformation [1,2], with applications ranging from soft tissues and biomaterials to elastomers and composites [3]. The imaging tools that enable DVC across various scales include magnetic resonance imaging [4], X-ray (micro) computed tomography [3, 5], ultrasound [6, 7], optical coherence tomography [8, 9], and confocal microscopy [10]. However, the existing DVC methods cannot directly resolve the spatial heterogeneity of mechanical properties in the 3D domain.

Elasticity imaging is a technique to reconstruct the spatial distribution of mechanical properties using available deformation and force measurements. In general, elasticity imaging is comprised of an inherently ill-posed mathematical problem because the stress distribution inside the domain is not available. Over the past three decades, many experimental, theoretical, and numerical studies in one- and two-dimensional geometries have tackled this topic, introducing various methods to solve the inverse problem [11, 12]. The 3D studies in the field have been relatively limited, however, mainly due to the prohibitive computational cost of these methods.

For 3D elasticity imaging, the existing approaches include direct methods, such as virtual fields methods, and domain decomposition methods, with varying levels of accuracy. Direct methods are known to have poor reconstruction accuracies when the material parameter field has sharp gradients [13, 14]. The virtual fields method relies on generating as many independent virtual loadings as the number of unknown parameters before solving the inverse problem in one pass [15]. However, this requirement is non-trivial to achieve for high-resolution 3D domains. Domain decomposition methods attempt to minimize an objective function that tracks the violation of constitutive compatibility in subdomains to identify stress terms first, followed by solving for elasticity parameters through kinematic measurements and constitutive equations. Domain decomposition in 3D has shown successful 3D reconstructions for domains with smooth elastic modulus gradients [16]. However, a common yet important drawback shared by these methods is that they have been mostly validated using non-complex geometries, e.g., a simple spherical inclusion inside a background.

While purely data-driven deep learning techniques have made advances in solving inverse material characterization problems, they have limited applicability in problems that fall outside the training dataset, hampering their generalizability. In recent years, methods that employ neural networks with physics-based loss functions to solve inverse problems have addressed some limitations of other conventional methods. In these methods, fully connected feed forward networks estimate parameter (and stress) fields given spatial coordinates as inputs. Respective loss terms corresponding to the physical equations, comprised of network outputs and other known parameters, are minimized to solve the inverse problem [17–20]. However, as with other methods’ computational cost limitations, neural network implementation for 3D inverse problems in elasticity imaging remains a challenge.

In this study, we use a modified version of El-UNet, an inversion physics-based neural network model that we previously developed based on the UNet encoder-decoder network [21], to solve inverse problems in linear isotropic and transversely isotropic elasticity in three dimensions. Our model estimates the material parameters by taking strain distributions as “input images” and enforcing boundary and domain physics loss functions. We present our novel findings in two main sections. First, we employ El-UNet to solve the 3D inverse heterogeneous isotropic linear elasticity problem on a brain tissue specimen with its complex geometrical features under compressive loading. Second, we employ a modified 3D El-UNet configuration in a transversely isotropic example of articular cartilage under compressive loading, where we estimate the space-dependent distribution of the five material parameters with given element orientations and data from three orthogonal uniaxial compressions. We quantify the corresponding reconstruction error and show superior performance compared to other approximations for 3D elasticity imaging.

## 2. Methods

### 2.1. Isotropic linear elasticity formulation

Elasticity constitutive equations in index notation is written as $\sigma_{ij}=C_{ijlm}\varepsilon_{lm}$, where σ and ε represent the stress and strain tensors and C is the fourth-order material properties tensor. This equation for isotropic linear elastic materials simplifies to

(1) $$\left\{\begin{array}{l} \sigma_{xx}\\ \sigma_{yy}\\ \sigma_{zz}\\ \sigma_{xy}\\ \sigma_{yz}\\ \sigma_{xz} \end{array}\right\}=\left[\begin{array}{cccccc} 2\mu +\lambda & \lambda & \lambda & 0 & 0 & 0\\ \lambda & 2\mu +\lambda & \lambda & 0 & 0 & 0\\ \lambda & \lambda & 2\mu +\lambda & 0 & 0 & 0\\ 0 & 0 & 0 & \mu & 0 & 0\\ 0 & 0 & 0 & 0 & \mu & 0\\ 0 & 0 & 0 & 0 & 0 & \mu \end{array}\right]\left\{\begin{array}{c} \varepsilon_{xx}\\ \varepsilon_{yy}\\ \varepsilon_{zz}\\ 2\varepsilon_{xy}\\ 2\varepsilon_{yz}\\ 2\varepsilon_{xz} \end{array}\right\}.$$

Where λ and μ are the Lamé parameters. Furthermore, the static equilibrium equations in the absence of body forces reduce to:

(2) $$\begin{array}{l} \frac{\partial \sigma_{xx}}{\partial x}+\frac{\partial \sigma_{xy}}{\partial y}+\frac{\partial \sigma_{xz}}{\partial z}=0,\\ \frac{\partial \sigma_{xy}}{\partial x}+\frac{\partial \sigma_{yy}}{\partial y}+\frac{\partial \sigma_{yz}}{\partial z}=0,\\ \frac{\partial \sigma_{xz}}{\partial x}+\frac{\partial \sigma_{yz}}{\partial y}+\frac{\partial \sigma_{zz}}{\partial z}=0. \end{array}$$

Finally, elastic modulus and Poisson’s ratio can be derived from the Lamé parameters using:

(3) $$\begin{array}{l} E=\frac{\mu (3\lambda +2\mu )}{\lambda +\mu },\\ v=\frac{\lambda }{2(\lambda +\mu )}. \end{array}$$

### 2.2. Transversely isotropic linear elasticity formulation

In this section, the prime notation denotes quantities in the local coordinate system for the transversely isotropic element where $x^{\prime }$ is the longitudinal axis and $y^{\prime }z^{\prime }$ is the transverse plane. Linear elasticity equations for transversely isotropic materials for a point in the space are:

(4) $$\left\{\begin{array}{c} {\sigma^{\prime }}_{xx}\\ {\sigma^{\prime }}_{yy}\\ {\sigma^{\prime }}_{zz}\\ {\sigma^{\prime }}_{xy}\\ {\sigma^{\prime }}_{yz}\\ {\sigma^{\prime }}_{xz} \end{array}\right\}=\left[\begin{array}{cccccc} {C^{\prime }}_{11} & {C^{\prime }}_{12} & {C^{\prime }}_{12} & 0 & 0 & 0\\ {C^{\prime }}_{12} & {C^{\prime }}_{22} & {C^{\prime }}_{23} & 0 & 0 & 0\\ {C^{\prime }}_{12} & {C^{\prime }}_{23} & {C^{\prime }}_{22} & 0 & 0 & 0\\ 0 & 0 & 0 & {C^{\prime }}_{44} & 0 & 0\\ 0 & 0 & 0 & 0 & \frac{{C^{\prime }}_{11}-{C^{\prime }}_{12}}{2} & 0\\ 0 & 0 & 0 & 0 & 0 & {C^{\prime }}_{44} \end{array}\right]\left\{\begin{array}{c} {\varepsilon^{\prime }}_{xx}\\ {\varepsilon^{\prime }}_{yy}\\ {\varepsilon^{\prime }}_{zz}\\ 2{\varepsilon^{\prime }}_{xy}\\ 2{\varepsilon^{\prime }}_{yz}\\ 2{\varepsilon^{\prime }}_{xz} \end{array}\right\}$$

Because the measured strain information and boundary conditions are in the global system orientation, the local parameters need to be transformed to the global state before constructing the constitutive equations in the global system. For this purpose, we assume the local coordinate system orientation at any point in the domain can be discovered by performing three consecutive rotations in space. These rotations include a rotation of ψ along the Z axis, followed by a rotation of θ around the now rotated $Y^{\prime }$ axis, and finally a rotation of φ around the $Z^{\prime \prime }$ axis.


(5) $$\begin{array}{l} Z=\left[\begin{array}{ccc} \cos(\psi ) & \sin(\psi ) & 0\\ -\sin(\psi ) & \cos(\psi ) & 0\\ 0 & 0 & 1 \end{array}\right],\\ Y^{\prime }=\left[\begin{array}{ccc} \cos(\theta ) & 0 & \sin(\theta )\\ 0 & 1 & 0\\ -\sin(\theta ) & 0 & \cos(\theta ) \end{array}\right],\\ Z^{\prime \prime }=\left[\begin{array}{ccc} \cos(\varphi ) & \sin(\varphi ) & 0\\ -\sin(\varphi ) & \cos(\varphi ) & 0\\ 0 & 0 & 1 \end{array}\right] \end{array}$$

The rotation matrix can be constructed by computing the multiplication of the matrices in Eq. (5).


(6) $$R=Z\times Y^{\prime }\times Z^{\prime \prime }.$$

Using this rotation matrix, R, each unit vector along the main axes of the global coordinate system can be transformed to its rotated state in the local coordinate system by

(7) $$u^{\prime }=R\times u$$

where u is a unit vector in the global coordinate system (x, y or z) and $u^{\prime }$ is the corresponding unit vector in the rotated system ($x^{\prime }$, $y^{\prime }$ or $z^{\prime }$). Accordingly, the transformation matrix, T, is defined as.


(8) $$T=\left[\begin{array}{lll} t_{11} & t_{12} & t_{13}\\ t_{21} & t_{22} & t_{23}\\ t_{31} & t_{32} & t_{33} \end{array}\right]=\left[\begin{array}{lll} \cos\left(x^{\prime },x\right) & \cos\left(x^{\prime },y\right) & \cos\left(x^{\prime },z\right)\\ \cos\left(y^{\prime },x\right) & \cos\left(y^{\prime },y\right) & \cos\left(y^{\prime },z\right)\\ \cos\left(z^{\prime },x\right) & \cos\left(z^{\prime },y\right) & \cos\left(z^{\prime },z\right) \end{array}\right]$$

where, $\cos\left(x^{\prime },x\right)$ represents the cosine of the angle between the global axis x and the rotated axis $x^{\prime }$. Using the terms of the T matrix, the Bond transformation matrix [22] is constructed as:

(9) $$B=\left[\begin{array}{cccccc} t_{11}t_{11} & t_{12}t_{12} & t_{13}t_{13} & t_{11}t_{12} & t_{12}t_{13} & t_{13}t_{11}\\ t_{21}t_{21} & t_{22}t_{22} & t_{23}t_{23} & t_{21}t_{22} & t_{22}t_{23} & t_{23}t_{21}\\ t_{31}t_{31} & t_{32}t_{32} & t_{33}t_{33} & t_{31}t_{32} & t_{32}t_{33} & t_{33}t_{31}\\ 2t_{11}t_{21} & 2t_{12}t_{22} & 2t_{13}t_{23} & t_{11}t_{22}+t_{12}t_{21} & t_{12}t_{23}+t_{13}t_{22} & t_{13}t_{21}+t_{11}t_{23}\\ 2t_{21}t_{31} & 2t_{22}t_{32} & 2t_{23}t_{33} & t_{21}t_{32}+t_{22}t_{31} & t_{22}t_{33}+t_{23}t_{32} & t_{23}t_{31}+t_{21}t_{33}\\ 2t_{31}t_{11} & 2t_{32}t_{12} & 2t_{33}t_{13} & t_{31}t_{12}+t_{32}t_{11} & t_{32}t_{13}+t_{33}t_{12} & t_{33}t_{11}+t_{31}t_{13} \end{array}\right]$$

The last step to compute the global stiffness matrix (C) from Eq. (1) using $C^{\prime }$ and B is

(10) $$C=B^{T}C^{\prime }B.$$

The equations can also be written in terms of five other independent parameters that are more physically relevant for fiber mechanics, i.e. longitudinal elastic modulus $\left(E_{\text{xx}}\right)$, transverse elastic modulus $\left(E_{\text{yy}}=E_{\text{zz}}\right)$, transverse plane Poisson’s ratio $\left(v_{yz}\right)$, transverse-longitudinal plane Poisson’s ratio $\left(v_{xy}=v_{xz}\right)$ and, transverse-longitudinal plane shear modulus $\left(G_{xy}=G_{xz}\right)$. The transverse plane shear modulus $\left(G_{xy}\right)$ is not an independent parameter itself and can be computed from other parameters. In the inverse implementation, the terms of the stiffness matrix from Eq. (4) get discovered, after which the following conversion equations are used to obtain the alternative representation of transversely isotropic elasticity parameters:

(11) $$\left\{\begin{array}{c} {E^{\prime }}_{\mathrm{xx}}\\ {E^{\prime }}_{\mathrm{yy}}\\ {v^{\prime }}_{xy}\\ {v^{\prime }}_{yz}\\ {v^{\prime }}_{xy}\\ {G^{\prime }}_{yz} \end{array}\right\}=\left\{\begin{array}{c} \frac{-2{{C^{\prime }}_{12}}^{2}+{C^{\prime }}_{11}{C^{\prime }}_{22}+{C^{\prime }}_{11}{C^{\prime }}_{23}}{{C^{\prime }}_{22}+{C^{\prime }}_{23}}\\ \frac{\left({C^{\prime }}_{22}-{C^{\prime }}_{23}\right)\left(-2{{C^{\prime }}_{12}}^{2}+{C^{\prime }}_{11}{C^{\prime }}_{22}+{C^{\prime }}_{11}{C^{\prime }}_{23}\right)}{{C^{\prime }}_{11}{C^{\prime }}_{22}-{{C^{\prime }}_{12}}^{2}}\\ \frac{{C^{\prime }}_{12}}{{C^{\prime }}_{22}+{C^{\prime }}_{23}}\\ \frac{{C^{\prime }}_{11}{C^{\prime }}_{23}-{{C^{\prime }}_{12}}^{2}}{{C^{\prime }}_{11}{C^{\prime }}_{22}-{{C^{\prime }}_{12}}^{2}}\\ {C^{\prime }}_{44}\\ \frac{\left({C^{\prime }}_{22}-{C^{\prime }}_{23}\right)}{2} \end{array}\right\}$$

The enforced static equilibrium equations remain the same as Eq. (2).

### 2.3. Compressive loading simulation of a heterogeneous medium

**Fig. 1.**
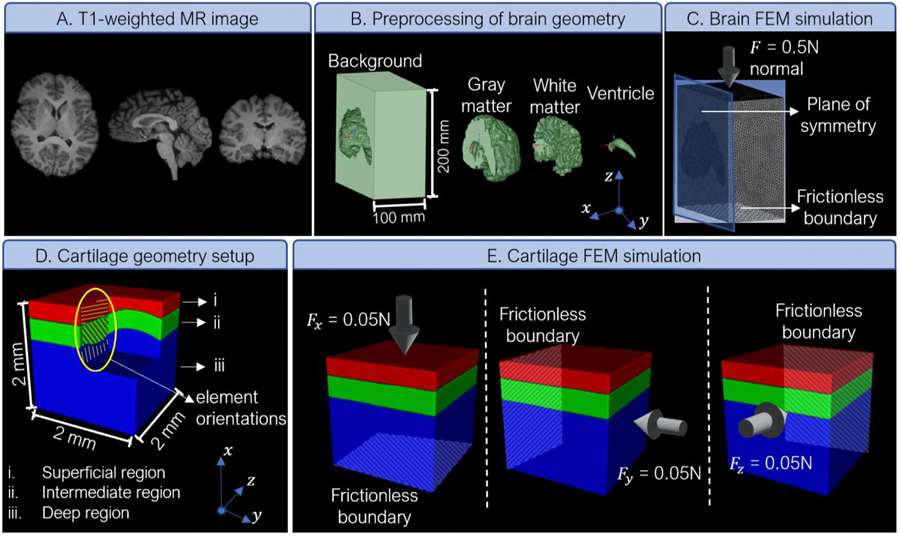
Details of synthetic data generation from elasticity imaging using finite element method (FEM) simulations for brain tissue (A-C) and cartilage (D, E).

Following our work on using brain tissue as a complex and biologically relevant specimen to test the elasticity imaging inversion methodology in two dimensions [20,21], we generated a synthetic 3D example by simulating the 3D deformation of brain tissue under compressive loading. To perform the simulation, first, we collected a T1 scan of the brain from a 28-year-old male using a 3.0 Tesla MRI scanner (Skyra, Siemens Healthcare, Germany) and a 32-channel head coil (human subject imaging approved by University of Arizona Institutional Review Board, February 2020). We used BrainSuite [23] to segment the T1-weighted image volume and exported masks for different regions of the brain (Fig. 1A). We simplified the segmentation by grouping deep brain structures under the white matter category. Therefore, the resulting geometry had three distinct regions: gray matter, white matter, and the ventricles. After manually refining some sharp edges of the geometry, we converted the masks to STL files using Slicer [24], and re-meshed them in InStep 3.0 (Solveering LLC, NM, USA) to reduce the number of triangles down to 10–15 % to simplify the finite-element (FE) modeling.

Next, we performed a finite element simulation of compressing a hydrogel cube containing the brain specimen. We imported the remeshed STL’s to SpaceClaim, part of the Ansys Workbench suite (Ansys, Inc, PA, USA), and created a solid model out of the surface geometries. Next, we made a model of the right hemisphere only, with a 200 mm×200 mm×100 mm background region around the brain and symmetry boundary condition at the midsagittal plane. We performed this step to reduce the computational load of the forward finite element simulation. We assigned material parameters to the gray matter, white matter, ventricles, and background according to Table 1 [20,21]. The ventricle’s properties were assigned the same as the background material. The geometry was discretized using tetrahedral mesh and uniaxial compressive loading was applied in the Z direction by a uniform force of 1 N on one side and frictionless boundary condition on the other. The other sides (other than the symmetry plane) were free boundaries. The loading (0.5 N) led to a maximum axial strain of about −0.035 in the domain. After running the simulation, we interpolated the unstructured data into a structured grid of 160×160×160 using the triangulation-based natural neighbor interpolation in MATLAB (MathWorks, MA). We exported the entire field by mirroring the results with respect to the sagittal plane of symmetry. As a separate experiment, to investigate the effect of input data noise on model estimations, we also included 5 % Gaussian noise in the input strains [25] (with respect to standard deviation of each source image) to contrast with the non-noisy input data example above.

### 2.4. Compressive loading of cartilage simulation

To test our model in discovering transversely isotropic material properties, we simulated compressive loading of an articular cartilage specimen. Articular cartilage consists of three distinct regions with different collagen fiber orientations and mechanical properties. Following the published anatomy and micromechanics of this tissue type [26–30], we developed a 2 mm×2 mm×2 mm geometry consisting of superficial, intermediate, and deep regions with distinct material parameters and fiber orientations (Table 1). For simplicity, we assumed that all the fibers (elements) in each region are elongated along one direction and assumed a continuum in which the fiber orientation is represented as the entire element orientation in the finite element analysis of the transversely isotropic material. We performed compressive loadings along the X, Y, and Z directions of the specimen separately, with constant force (0.05 N) on one side and frictionless boundary on the opposite side. Discovering the spatial distribution of all five material parameters would not be possible with only one loading as that only provides three equilibrium equations per voxel. Similar to the previous section, we included 5 % Gaussian noise [25] (with respect to standard deviation of each source image) in the input strains to have an identical example that includes noise in the input data.

### 2.5. 3D El-UNet implementation for isotropic linear elasticity

**Fig. 2.**
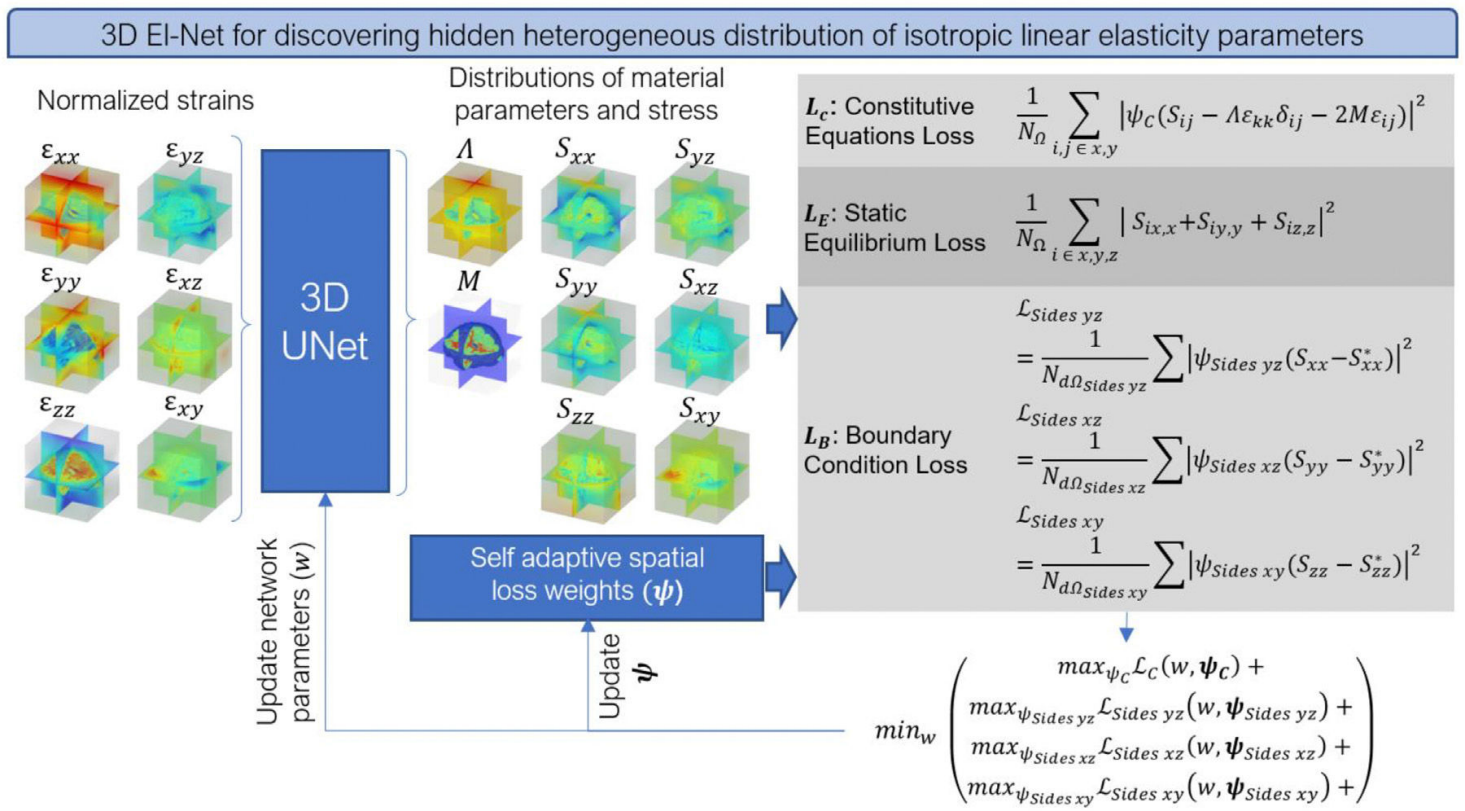
3D El-Net for estimating heterogeneous distribution of isotropic linear elasticity parameters.

For solving the inverse problems, we expanded our previously published physics-informed model El-UNet [21] – an encoder-decoder structure based on the original UNet architecture [31] – to solve inverse elasticity problems in 3D domains (Fig. 2). In brief, the neural network works as an operator taking in strain fields in 3D as input and returning stress and material parameters as outputs. The proposed physics-informed UNet solves one problem at a time by satisfying the physical equations and boundary conditions governing the problem, as opposed to data-driven models that get trained on numerous problems to infer a solution on an unseen example later. We used a three-level deep UNet for both examples in this work consisting of double convolutions with 64 and 128 channels per depth moving in the downward path, and 256 channels in the bottleneck stage, and the reverse trend for the upward path. The network takes in a 6-channel input, each channel containing the volumetric distribution of a strain tensor term (three normal and three shear terms) and estimates eight volumetric distributions: first and second dimensionless Lamé parameters (Λ and M, uppercase notation to indicate dimensionless parameters) along with three normal and three shear stress distributions. The algorithm then uses the isotropic linear elasticity constitutive loss equation, static equilibrium loss equation, and boundary condition loss equations, with self-adaptive spatial weights applied to constitutive and boundary condition equations according to our previous work [21]. The partial derivatives in the static equilibrium equations are computed using finite central difference approximation. We used the Adam optimizer with a learning rate of 0.001 to minimize the loss value and trained the model for 40,0 0 0 epochs on an Nvidia v100 GPU. El-UNet was not given prior knowledge about the symmetrical conditions of the domain, but the sign for partial derivatives of stress with an X component for the mirrored side had to change when implementing static equilibrium loss equations. In addition to evaluating final reconstruction accuracy for E and v, we also obtained relative stiffness maps by normalizing the elastic modulus and axial strain distributions using the maximum value in each field. To investigate the plane stress/strain assumptions for approximating a fully 3D problem, we used 2D El-UNet with plane stress and plane strain assumptions on five coronal slices of the volume spaced apart by 25 mm increments to compare accuracy of reconstructions with the 3D solution.

### 2.6. 3D El-UNet implementation for transversely isotropic linear elasticity

**Fig. 3.**
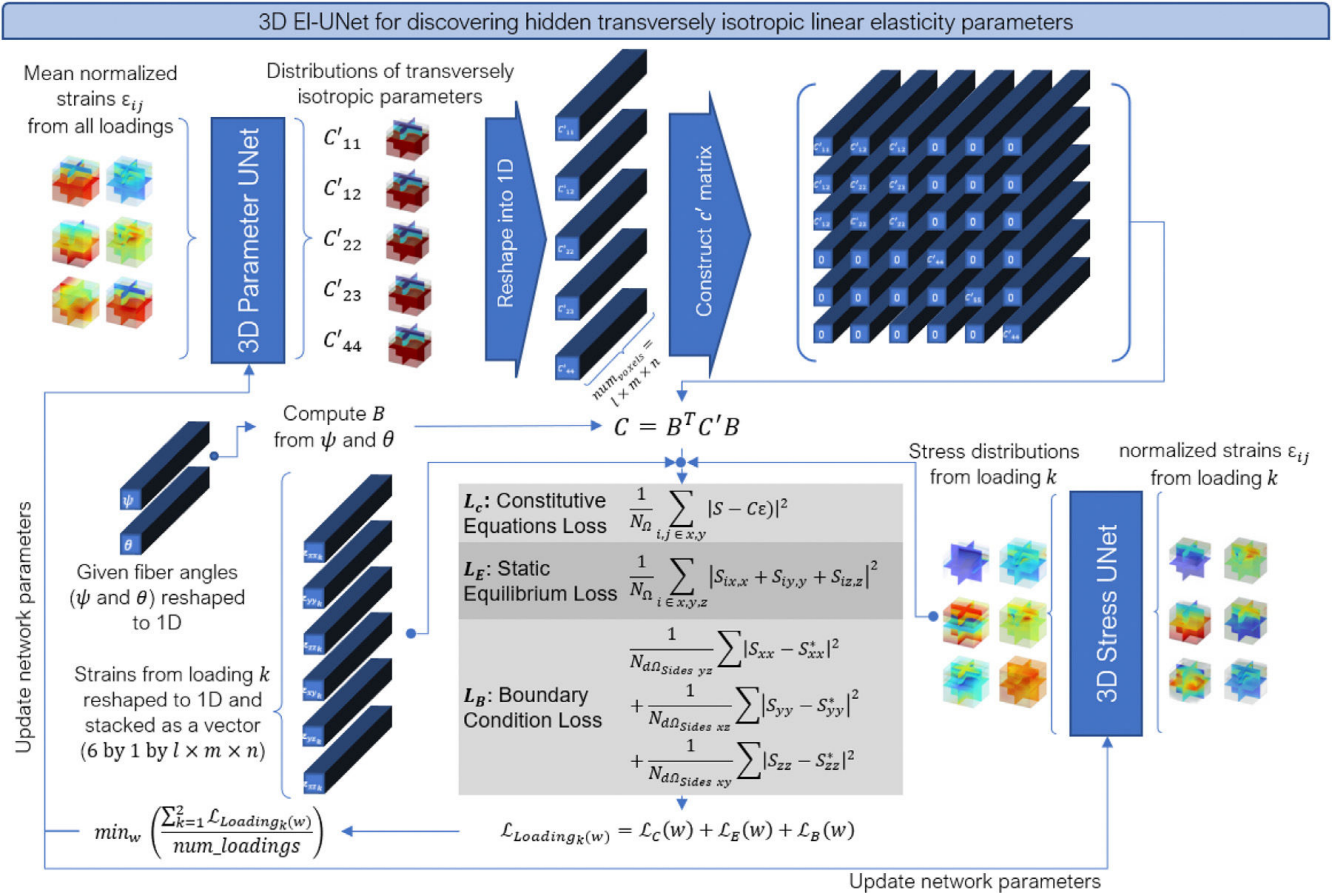
3D El-UNet for estimating heterogeneous distribution of transversely isotropic linear elasticity parameters.

We implemented a modified variation of 3D El-UNet for the transversely isotropic parameter estimation problem (Fig. 3). The network structure of the model remained the same as the isotropic elasticity El-UNet, however we used two UNets taking different inputs for this problem. The input channels for the first UNet, named Parameter UNet, consist of mean normalized strains from all X, Y, and Z loadings of the specimen, resulting in six volumetric channels in total. The output consists of five volumetric channels, representing the five material parameters fully defining transversely isotropic linear elasticity in the element direction (local orientation of fibers). These volumetric channels are reshaped to 1D arrays before populating the local stiffness matrix for each voxel, being stacked in the batch direction, creating a n-by-6-by-6 stiffness matrix, where n is the total number of voxels in the volume. Next, using the given fiber angles, we compute the Bond transformation matrix using Eqs. (5–9), and compute the stiffness matrix in the global orientation using Eq. (10), leading to another n-by-6-by-6 matrix. The proposed structuring of the B and $C^{\prime }$ matrices enables efficient parallelized computation of matrix multiplication in the batch dimension (n). The other UNet, named Stress UNet, takes in normalized strains from a single loading and outputs estimated stress distributions. Finally, for each loading, first, the stress tensor is calculated by plugging in strain values, and fiber directions and material parameters (Output of Parameter UNet). The balance between these stress values and the output of the Stress UNet forms the constitutive equations loss. This loss is summed up with static equilibrium and boundary condition losses. This step is performed for the X, Y, and Z loadings separately, and the mean of the total loss from these three cases is calculated to perform the backpropagation and update of network parameters.

## 3. Results

### 3.1. 3D El-UNet resolves linear elastic parameter distributions with high accuracy

**Fig. 4.**
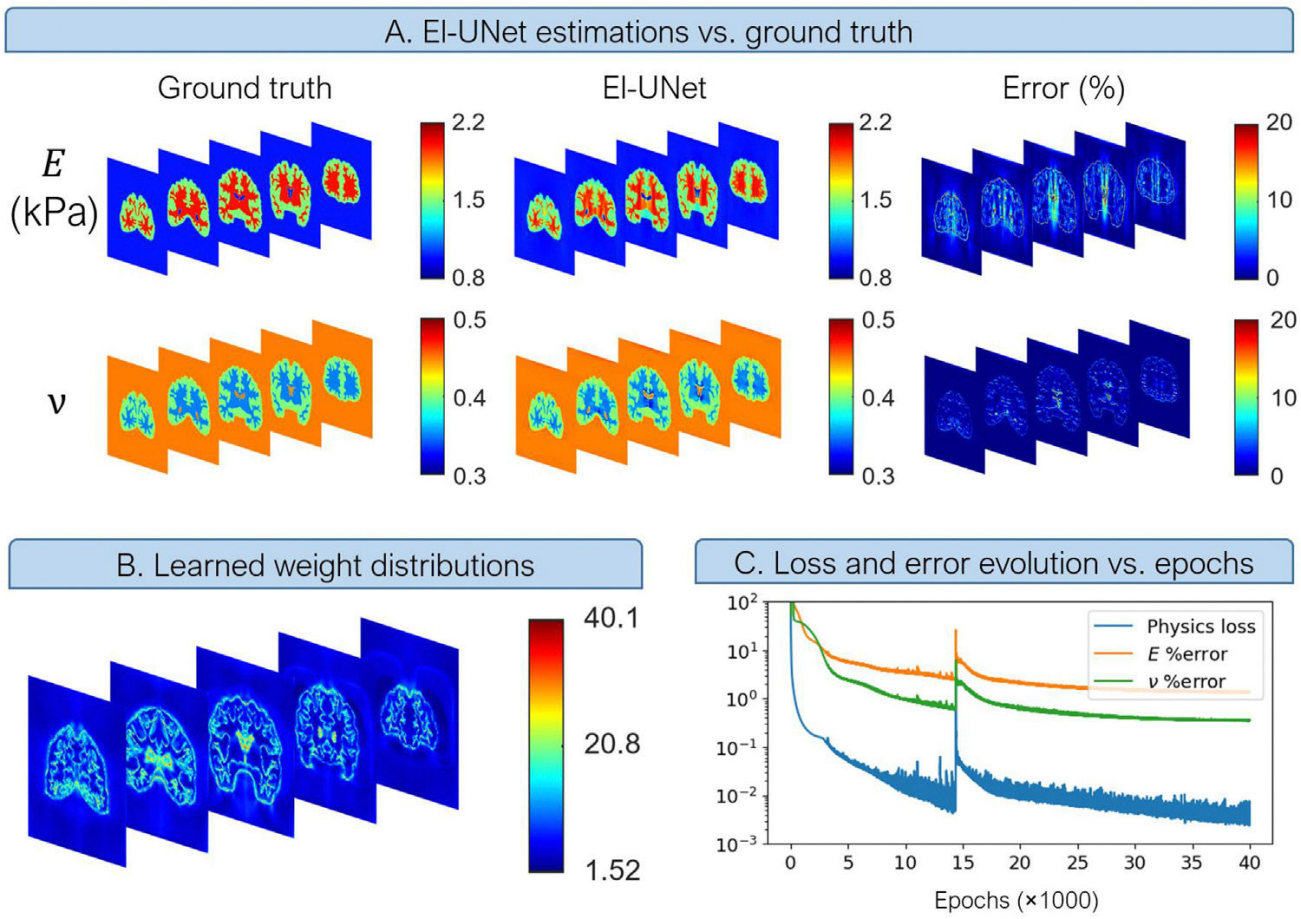
El-UNet estimation of isotropic linear elasticity parameters for a simulated brain tissue under compressive uniaxial loading. A) The estimations closely follow ground truth absolute values and spatial patterns. B) The self-adaptive spatial weighting algorithm learns to penalize the model more near the domain’s geometrical complexities. C) After 40,0 0 0 training epochs the relative error rates for E and v fall to under 1.5 %.

We tested the robustness of the isotropic linear elasticity El-UNet for a simulated imaging experiment of brain tissue under quasistatic uniaxial compression and investigated the performance in terms of estimation accuracy. El-UNet discovered the embedded brain shape and the corresponding regional elastic modulus and Poisson’s ratio estimations with high accuracy (Fig. 4A). The error distributions reveal that the elastic modulus estimation had relatively higher errors inside the brain tissue, with an overall mean absolute relative error of 1.33 % for elastic modulus and 0.33 % for Poisson’s ratio. This UNet implementation benefitted from self-adaptive weighting of constitutive and boundary equations, with the former showing increased weighting learned through solving the inverse problem for areas in and around the brain, especially around the transition zones (Fig. 4B). The evolution of total physics loss and elasticity parameter estimation errors across epochs reveal a steep drop of all these values for the first 50 00 epochs, where the estimation errors fall below 10 % (Fig. 4C). Estimations using noisy input strains expectedly showed higher errors, but largely discovered the complex patterns in the images (Supplementary Figure S1). Mean absolute relative errors for the model using noisy strains were 4.327 % for elastic modulus and 1.32 % for Poisson’s ratio.

### 3.2. 3D El-UNet produces relative stiffness distributions highly agreeing with ground truth

**Fig. 5.**
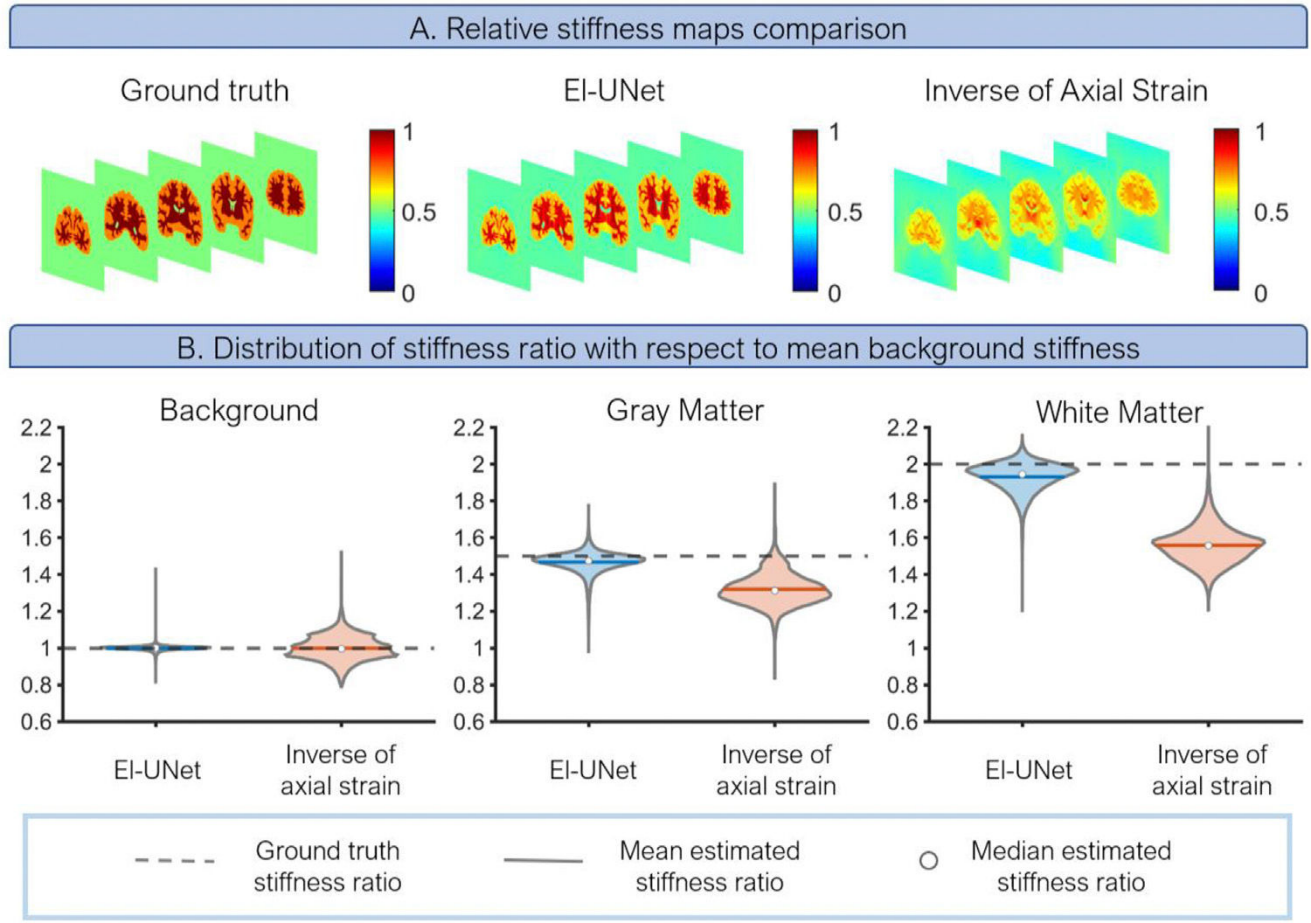
Comparison of El-UNet with inverse of axial strain as a method to plot relative stiffness distributions. A) The relative stiffness map obtained from El-UNet estimations clearly distinguishes the different regions in the image while the inverse of axial strain misses the geometrical complexities. Ground truth and El-UNet estimations are normalized with respect to the maximum stiffness value in their respective fields whereas the inverse of axial strain map is obtained by normalizing with respect to maximum inverse axial strain. B) Comparing the statistical distribution of stiffness ratios across the various regions in the volume against ground truth values demonstrates the considerable improvement gained by El-UNet over the axial strain method.

We generated relative stiffness maps from the 3D El-UNet solution and the conventional axial strain $\left(\varepsilon_{zz}\right)$ field to compare our method to a simple reconstruction used in 3D elasticity imaging. Relative stiffness maps of the El-UNet estimation reveal advantages gained by fully solving the inverse problem compared to the simplified inversion of axial strain assumption (Fig. 5). In terms of distinguishing between the three distinct material types in this example, El-UNet almost fully replicated the ground truth level of reconstruction, while the inverse of axial strain failed to capture the boundaries of different regions and did not distinguish between white and gray matter (Fig. 5A). Quantifying the relative stiffness ratios with respect to the background shows that gray matter and white matter estimations from El-UNet are much closer to expected ground truth ratios (Fig. 5B). The background distribution of stiffness ratios indicates that while the inverse of axial strain results cluster around two points around the ground truth value of 1, the El-UNet results cluster around 1, indicating superior performance.

### 3.3. 3D El-UNet produces more accurate reconstructions than plane strain and plane stress solutions

**Fig. 6.**
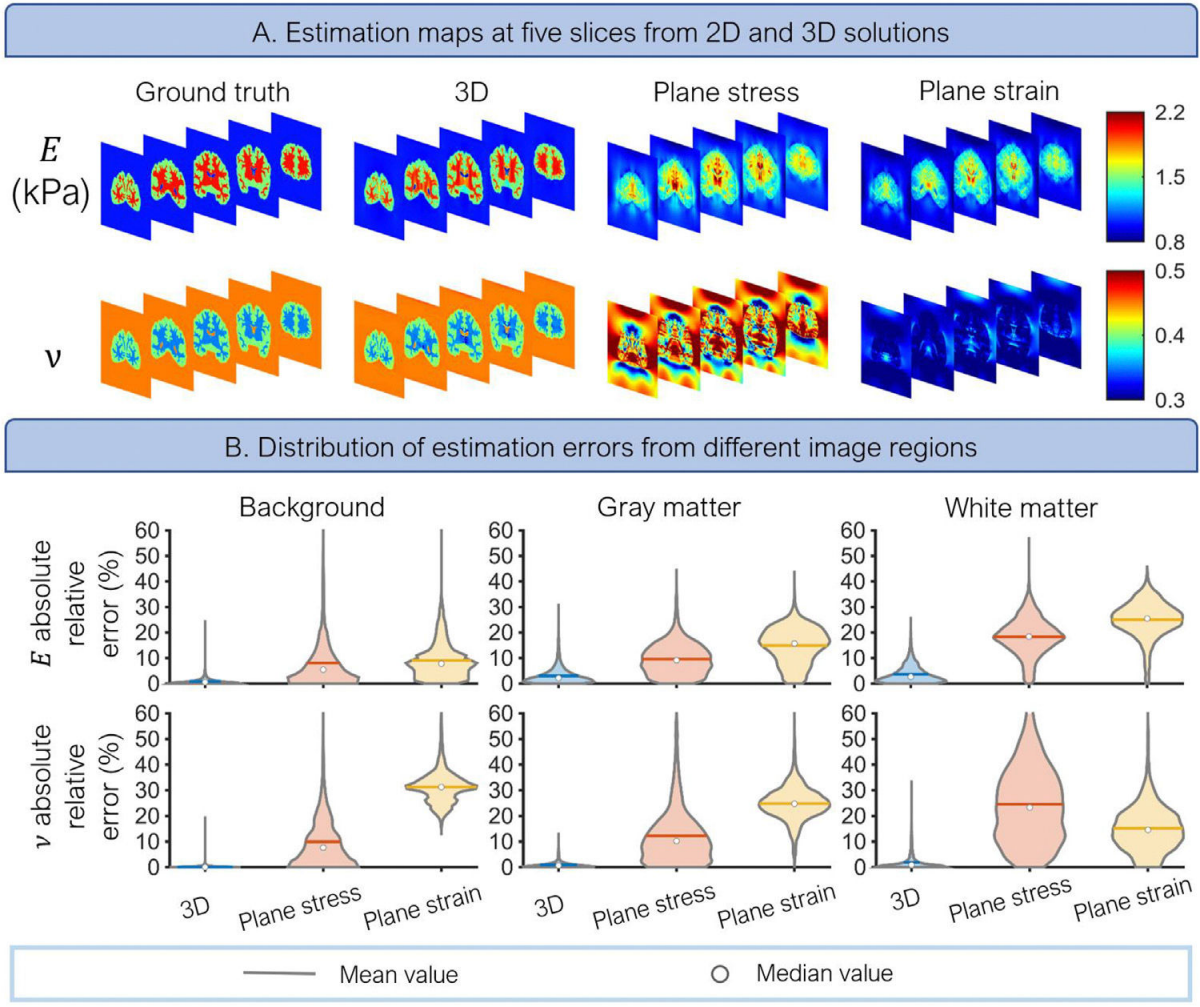
Comparison of 3D El-UNet with 2D El-UNet used for slices from the 3D volume using plane stress and plane strain assumptions. Both visualized maps and quantified error distributions reveal the accuracy gained by solving the fully 3D problem using El-UNet.

We compared the performance of the 3D El-UNet to solve the inverse 3D problem to using 2D El-UNet with plane stress and plane strain assumptions on coronal slices to evaluate the gain in accuracy of reconstructions in all regions of the image. Visualized results demonstrate the sharp contrast achieved in parameter maps from the 3D solution, while plane stress and plane strain assumptions fail to capture the complex pattern inside the brain (Fig. 6A). Quantified analysis of reconstruction error for different regions in the image also demonstrates the superiority of 3D solution compared to 2D approximations for the five slices under study (Fig. 6B). The 3D configuration results in mean absolute relative errors of under 5 % for either elasticity parameter on all regions. However, plane stress and plane strain assumptions lead to larger errors (> 10 % mean value) for gray matter and white matter reconstructions. Additionally, 2D reconstructions lead to a wider spread of errors.

### 3.4. Five transversely isotropic elasticity parameters are spatially discovered using information from three perpendicular loadings

**Fig. 7.**
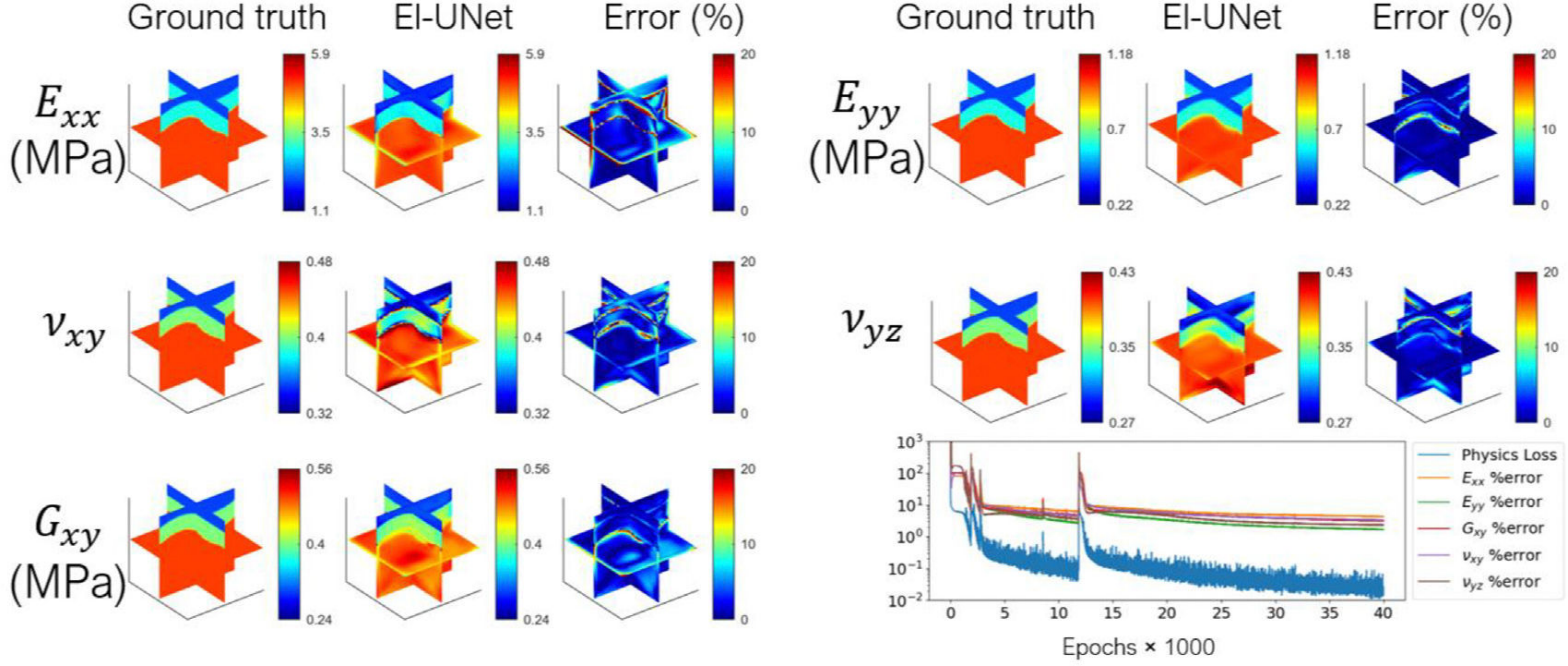
El-UNet results for reconstruction of heterogeneous transversely isotropic elasticity parameters. El-UNet discovers the zones having different material parameters and estimates the parameters with high accuracy and errors under 5 %.

We used the El-UNet model to solve for elasticity parameters in a simulated experiment of an articular cartilage specimen consisting of heterogeneous distribution of fibers and elasticity parameters under three uniaxial loading states (Fig. 7). This scenario shows the ability of the proposed El-UNet in discovering heterogeneous fiber mechanics distributions. The results indicate highly accurate reconstruction of all five unknown elasticity parameters (Mean estimation errors: $E_{\text{xx}}:4.28\;\%$, $E_{\text{yy}}:1.66\;\%$, $v_{xy}:3.30\;\%$, $v_{yz}:2.33\;\%$, $G_{xy}:3.11\;\%$). Similar to the isotropic estimations for the brain loading example, estimations using noisy input strains showed higher errors, but discovered the layers in the domain as well as their parameter fields (Supplementary Figure S2). Mean estimation errors from the noisy strains were: $E_{\text{xx}}:8.91\;\%$, $E_{\text{yy}}:3.81\;\%$, $v_{xy}:7.43\;\%$, $v_{yz}:4.67\;\%$, $G_{xy}:4.82\;\%$.

## 4. Discussion

We proposed a physics-informed UNet-based neural network model to estimate the 3D spatial distribution of isotropic and transversely isotropic linear elastic material properties given the strain fields and normal stress boundary data. In this approach, the network learns the material parameters by taking in normal and shear strain distributions and enforcing constitutive equations, static equilibrium, and normal stress boundary conditions at the six sides of the geometry. In contrast with data-trained neural network models, the physics-informed El-UNet does not have access to the ground truth material parameter and stress fields in the domain, and simply finds those by attempting to meet the constraints of the governing equations. More generally, our work extends current physics-informed elasticity imaging to three dimensions while ensuring reasonable computation time. It also provides a minimally invasive mechanical characterization method for deformable materials. Our approach has the potential to aid diagnostics, *in vitro* or *ex vivo* tissue characterization, and defect analysis.

Typical applications of digital volume correlation are mostly limited to strain distributions as surrogates for a stiffness measure. This simplification does not provide any quantitative measure of mechanical properties and only produces a relative stiffness map, obtained by inversing the strain map and normalizing with respect to maximum inverse strain. However, as we show in this study, the relative stiffness distribution does not provide enough contrast to distinguish between the different heterogeneous regions in the specimen. On the other hand, the El-UNet solution of the inverse problem simultaneously captures the distribution of elastic modulus and Poisson’s ratio from the strain distributions and the normal-to-surface stress boundary conditions.

We present a method for full mechanical characterization of two biologically relevant examples in a minimally invasive manner. The brain tissue is comprised of many distinct regions and the conventional method for mechanical characterization is dissection into small pieces and performing separate mechanical tests, affecting tissue properties in handling and long testing times [32–35]. Combining 3D full field measurement of deformation under loading coupled with using an inversion model such as El-UNet enables discovery of material parameter distribution in larger samples without having to dissect into small pieces. The transversely isotropic example on the other hand demonstrates the capability of the model to discover tissue heterogeneities across five different parameters using simple compressive loadings. Directly identifying any of these parameters requires specific mechanical testing designs that isolate the effect of a given material parameter.

The transversely isotropic example presented in our study demonstrates a feasible approach for inversion in multi-parameter material models. A drawback for quasistatic elasticity imaging scenarios is that the number of unknown parameters should be equal or smaller than the number of available static equilibrium equations, which is two for 2D and three for 3D examples from a single loading state. Whereas simple material models such as linear isotropic elasticity and neoHookean hyperelasticity have less than three parameters, anisotropic linear elasticity and most other hyperelastic models have equal to or more than three parameters. Therefore, to adequately inform the physics-informed deep learning model for the five-parameter transversely isotropic example in our study, we performed three compressive loading simulations in orthogonal directions and enforced the mean loss of the three states to train the neural network. This modification effectively increased the number of available equilibrium equations from three to nine, enabling the estimation of the five unknown parameters. We experimented with two and three loading states and learned that while two provides sufficient information to avoid having an under-determined problem, having three states ensures better quality across ideal and non-ideal (noisy) input data. In summary, the transversely isotropic El-UNet uses the compressive normal stresses on the loaded boundaries along with strain data from this combined loading to estimate all elasticity parameters by having known fiber orientations in the model.

The linearized formulation of constitutive equation ensures convergence to ground truth values for all parameters of the two material models in the current study. We observe through our experimentation with isotropic linear elasticity that the unknown parameters are discovered more accurately when the formulation is linear with respect to the parameters that are the outputs of the network. In our previous work, we used Lamé parameters for plane strain and plane stress followed by necessary conversions once the run finished to obtain elastic modulus and Poisson’s ratio [20, 21]. The parameters of interest can then be computed using predefined or derived conversion equations. We employ the same analogy to the transversely isotropic example by estimating intermediary parameters that the constitutive equations remain linear with respect to and convert to physically relevant parameters after the model converges. The reason for this unique behavior could be the following. When the parameters that are the output of the network have non-linear contributions in the equations, slight changes in the output of the network from each update might lead to large perturbations in the loss function, effectively leading the model away from the global minimum of the optimization problem. Unlike iterative finite element approaches, where each forward update of the model based on a new distribution of parameters results in a complete solution of the problem for that distribution, each forward pass of the neural nets is simply an iteration of the model with its updated weights and not necessarily the converged solution.

We also investigated the performance of the proposed models under non-ideal data quality conditions with input strain images having 5 % Gaussian noise. Because inverse elasticity algorithms based on neural network architectures as function approximators or operators involve first-order derivatives acting on stress values, they can potentially amplify the noise in the estimated distributions, as we have demonstrated previously [21]. However, our proposed implementations have an internal regularization step that balances between stress estimations obtained via two different paths. The first path yields stress values that are calculated using constitutive equations with strains in addition to material parameters that are neural network outputs. The second path produces stress estimations as output of a neural network directly. Minimizing the mean squared error loss between these two stress outputs (while minimizing the summation of all physics and boundary losses) internally denoises estimated stress fields and consequently mitigates the noise amplification in the static equilibrium loss. While our approach was mostly effective in mitigating noise effects, further innovations can improve model robustness against imperfect input data.

Several aspects of our study can be improved in the future. While our study shows significant promise for solving the inverse mathematical problem, the inversion model requires further development to show robustness in data obtained in experimental and potentially clinical settings. The strain distributions can be obtained from digital image correlation while normal boundary stress distribution can either be measured using pressure mapping technology or derived by using a hydrogel with known properties as the background material. The next area that can be developed in future models is inclusion of more complex material models such as hyperelasticity or viscoelasticity. We anticipate that some strategies that we present in this study, including informing the model on multiple loadings and leveraging linearized formulation, can pave the way for using these physics-informed implementations for discovery of hyperelastic and viscoelastic material parameters from quasi-static and dynamic loadings. As a technical challenge, we trained the model on the entire volume and needed high-memory GPU units (16–32 GB memory) to meet the demand from this large dataset. Strategies to train the network in minibatches of data can be developed to tackle this problem in the future. Finally, in *in vivo* experiments, it is challenging to quasi-statically load tissues such as the brain or cartilage as presented in this study. Other methods, such as MRI elastography (MRE) and shear wave elastography, with certain assumptions yield estimations for physically relevant material parameters of the brain tissue. The benefits and drawbacks of inversion methods that are conventionally used should be weighed against neural network-based solutions. For example, the power of the neural networks as operators can also potentially be leveraged in a physics-informed dynamic inverse elasticity problem. The implementation of this method for dynamic loading conditions and comparison with existing alternatives are potential next steps of the current work.

## Supplementary Material

Appendix

example

figures

tables

Supplementary material associated with this article can be found, in the online version, at doi:10.1016/j.actbio.2024.06.038.

## Figures and Tables

**Table 1:** Assigned material properties for finite-element modeling of loaded specimens.

Material			Material Parameters			

Isotropic linear elastic example: Brain						

			E (kPa)			v

White Matter			2			0.35
Gray Matter			1.5			0.4
Ventricles/Background			1			0.45
Transversely isotropic linear elastic example: Articular Cartilage						

	Fiber angle (deg)	$E_{\text{xx}}$ (MPa)	$E_{\text{yy}}$ (MPa)	$G_{\text{xy}}$ (MPa)	$v_{\text{xy}}$	$v_{\text{yz}}$

Superficial region	35	2	0.4	0.3	0.35	0.3
Intermediate region	60	3	0.6	0.4	0.4	0.35
Deep region	80	5	1	0.5	0.45	0.4

## Statement of significance

Elasticity imaging is a technique that reconstructs mechanical properties of tissue using deformation and force measurements. Given the complexity of this reconstruction, most existing methods have mostly focused on 2D problems. Our work is the first implementation of physics-informed UNets to reconstruct three-dimensional material parameter distributions for isotropic and transversely isotropic linear elastic materials by having deformation and force measurements. We comprehensively validate our model using synthetic data generated using finite element models of biological tissues with high bio-fidelity–the brain and articular cartilage. Our method can be implemented in elasticity imaging scenarios for *in vitro* and *ex vivo* mechanical characterization of biomaterials and biological tissues, with potential extensions to *in vivo* diagnostics.
