# Supplementary material for: Discovering 3D hidden elasticity in isotropic and transversely isotropic materials with physics-informed UNets
Source: Acta Biomater. Author manuscript; Available in PMC 2026 Apr 29. (PMC13128258; doi:10.1016/j.actbio.2024.06.038)
Supplement: Appendix [file NIHMS2148087-supplement-Appendix.docx]

**Supplementary Materials**


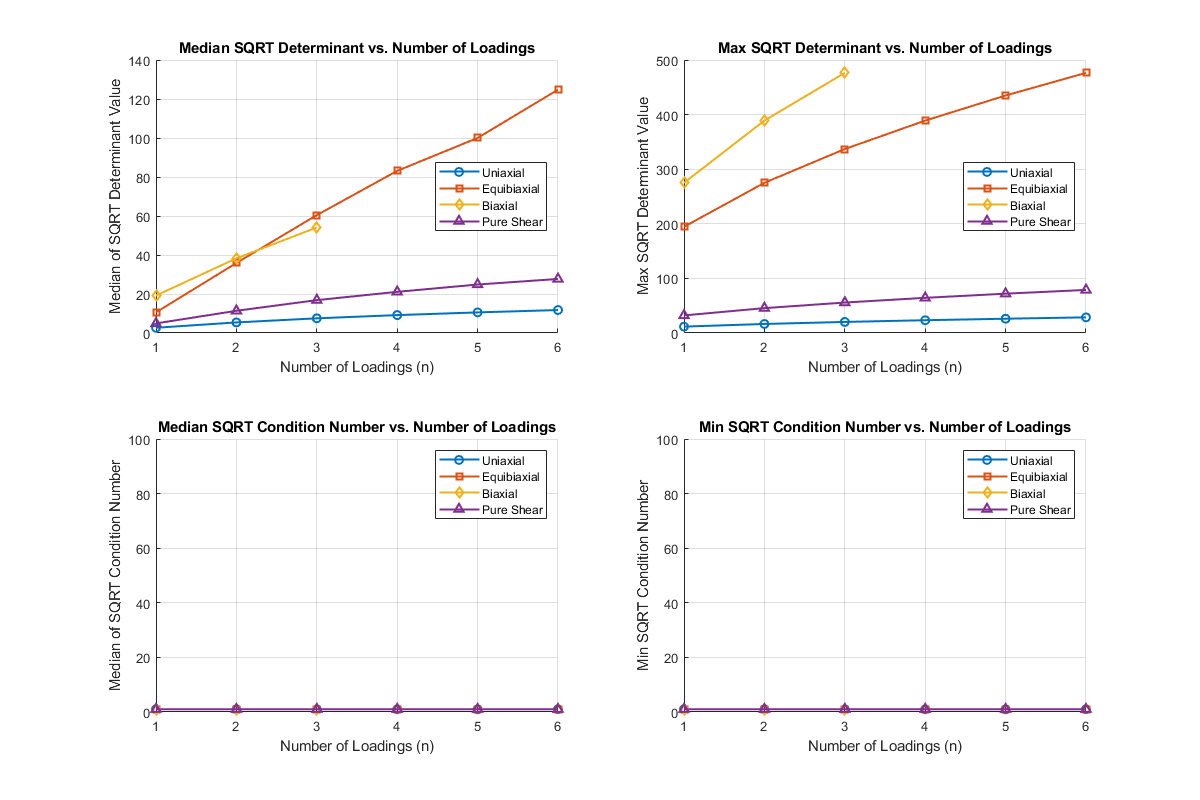


**Figure S1.** Max and median of SQRT determinant, and min and median SQRT condition number vs the number of measurements for Neo-Hookean model and stretch ranges between 0.4 and 1 (i.e., up to 60% compression) for different loading modes.


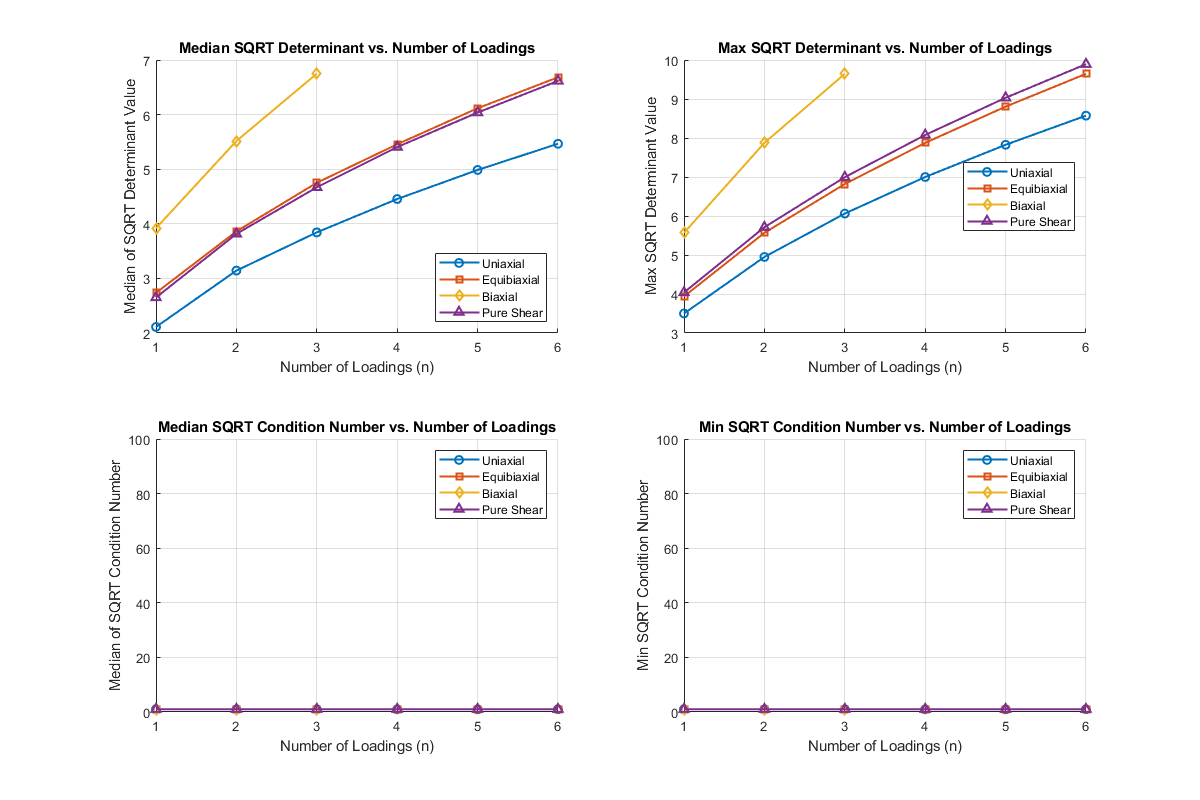


**Figure S2.** Max and median of SQRT determinant, and min and median SQRT condition number vs the number of measurements for Neo-Hookean model and stretch ranges between 1 and 2 (i.e., up to 100% stretch) for different loading modes.


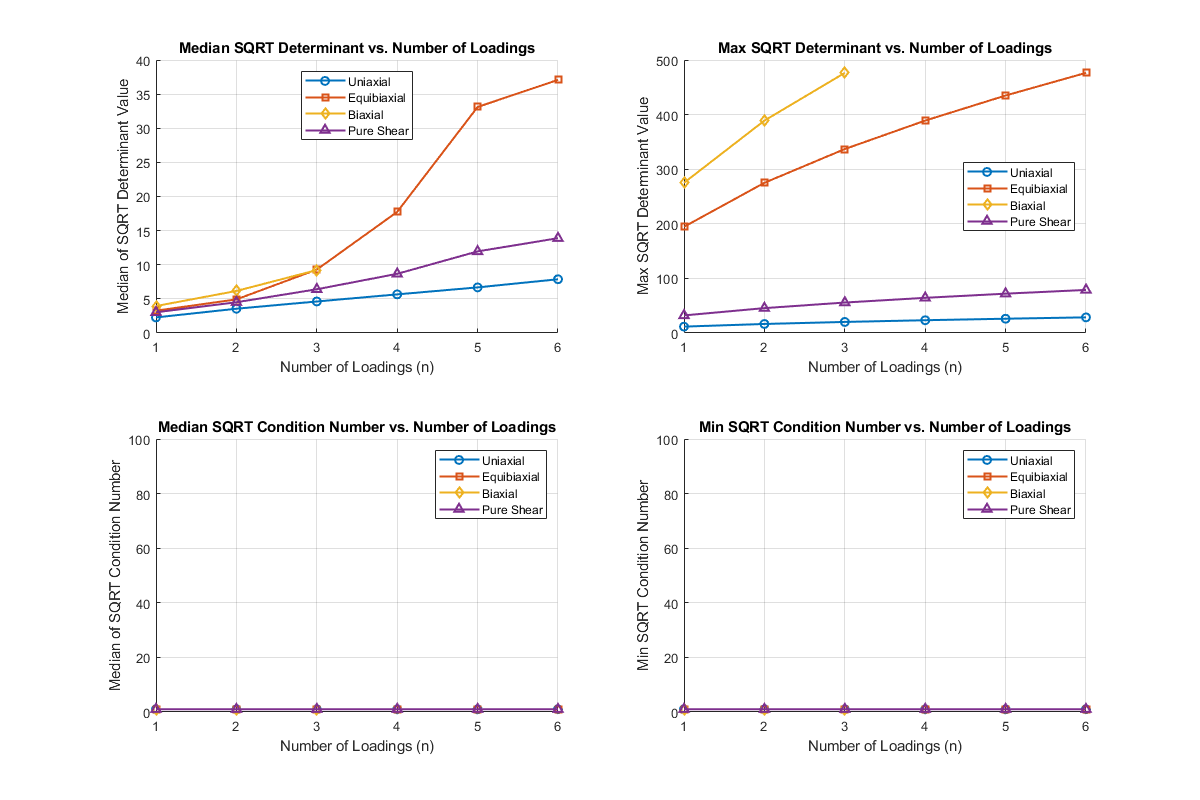


**Figure S3.** Max and median of SQRT determinant, and min and median SQRT condition number vs the number of measurements for Neo-Hookean model and stretch ranges between 0.4 and 2 (i.e., both tension and compression) for different loading modes.


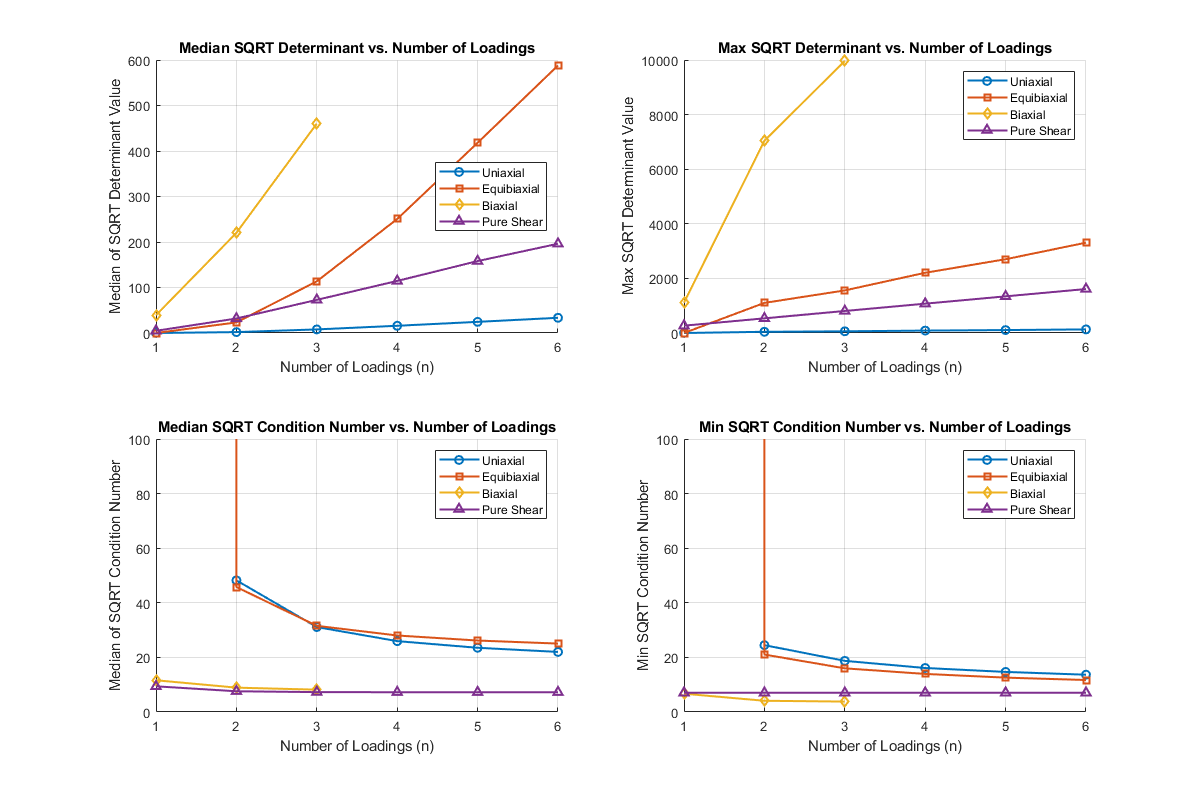


**Figure S4.** Max and median of SQRT determinant, and min and median SQRT condition number vs the number of measurements for Mooney-Rivlin model and stretch ranges between 0.4 and 1 for different loading modes.


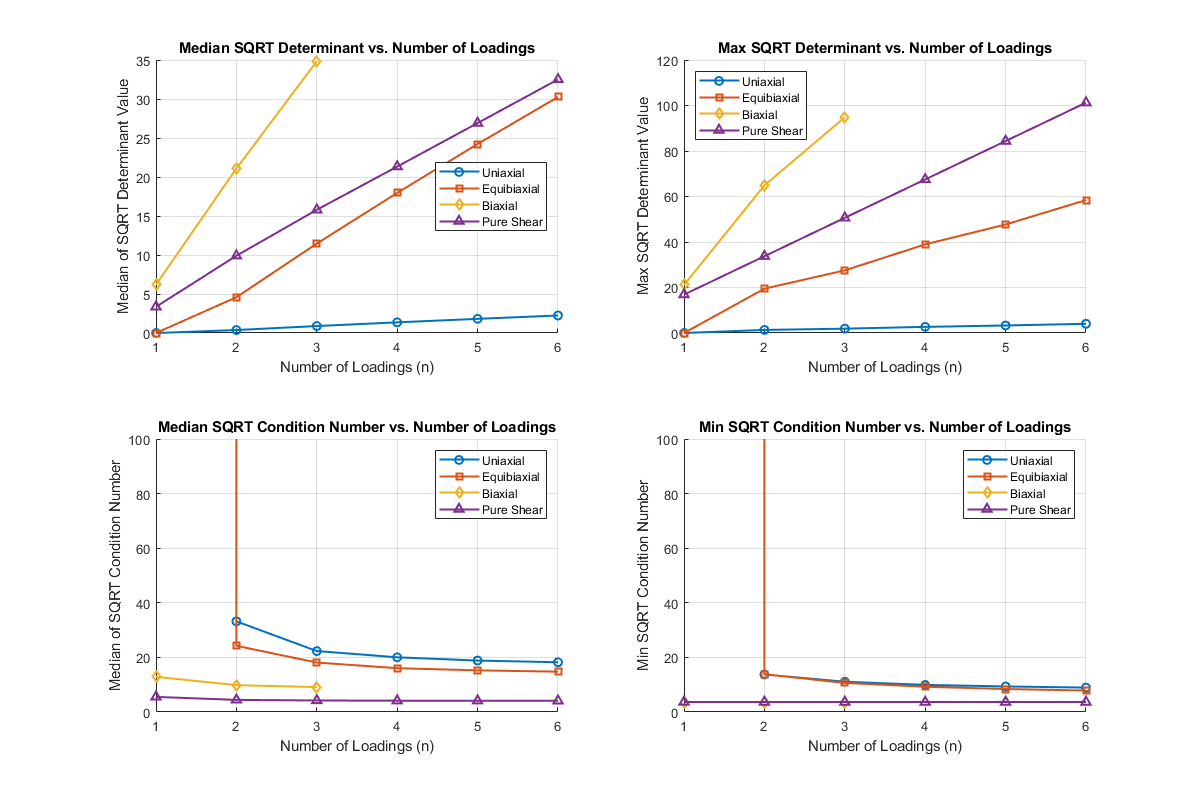


**Figure S5.** Max and median of SQRT determinant, and min and median SQRT condition number vs the number of measurements for Mooney-Rivlin model and stretch ranges between 1 and 2 for different loading modes.
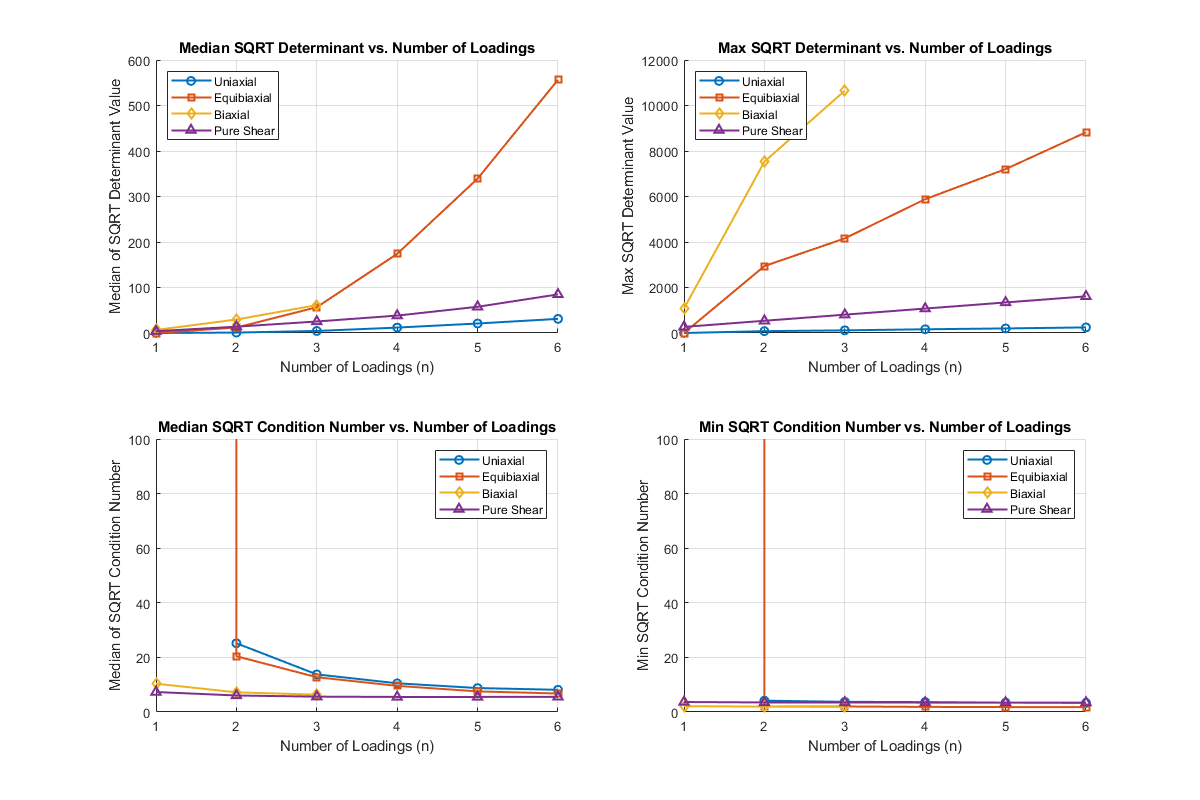


**Figure S6.** Max and median of SQRT determinant, and min and median SQRT condition number vs the number of measurements for Mooney-Rivlin model and stretch ranges between 0.4 and 2 for different loading modes.


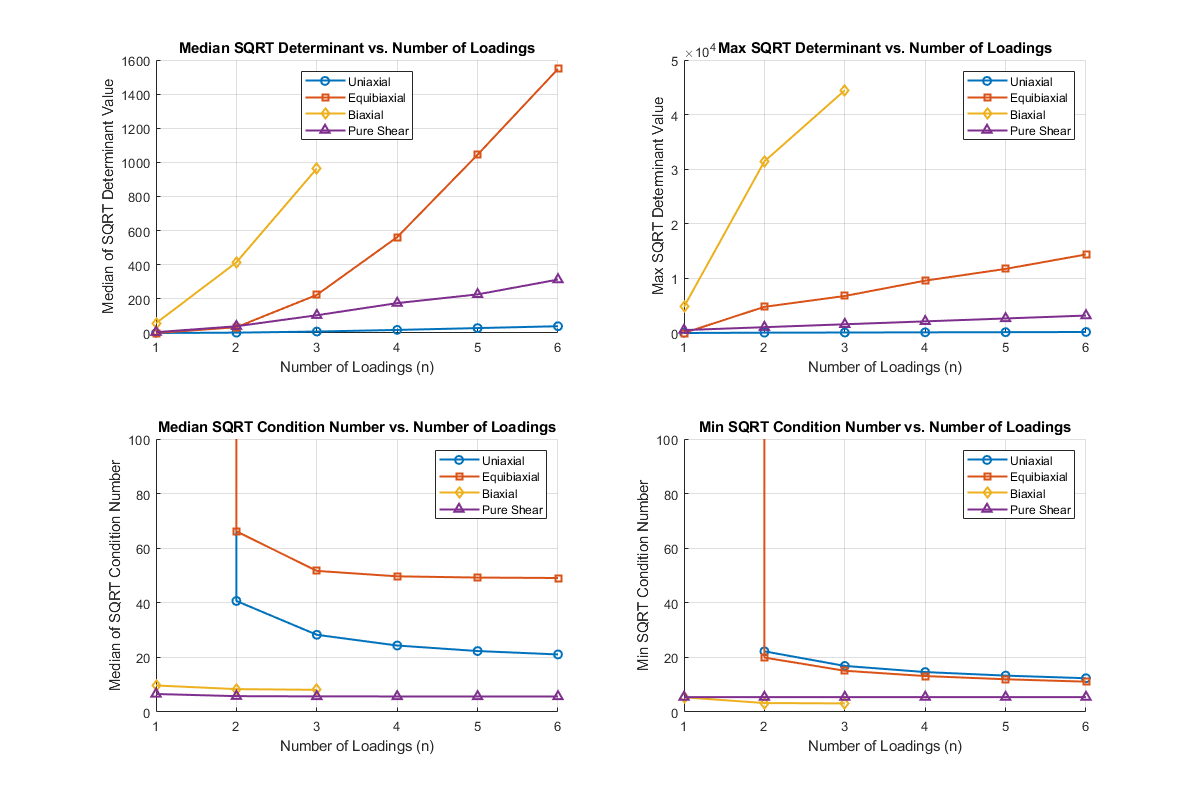


**Figure S7.** Max and median of SQRT determinant, and min and median SQRT condition number vs the number of measurements for Ogden model with $N=2, \alpha_{1}=3, \alpha_{2}=-3$ and and stretch ranges between 0.4 and 1 for different loading modes.


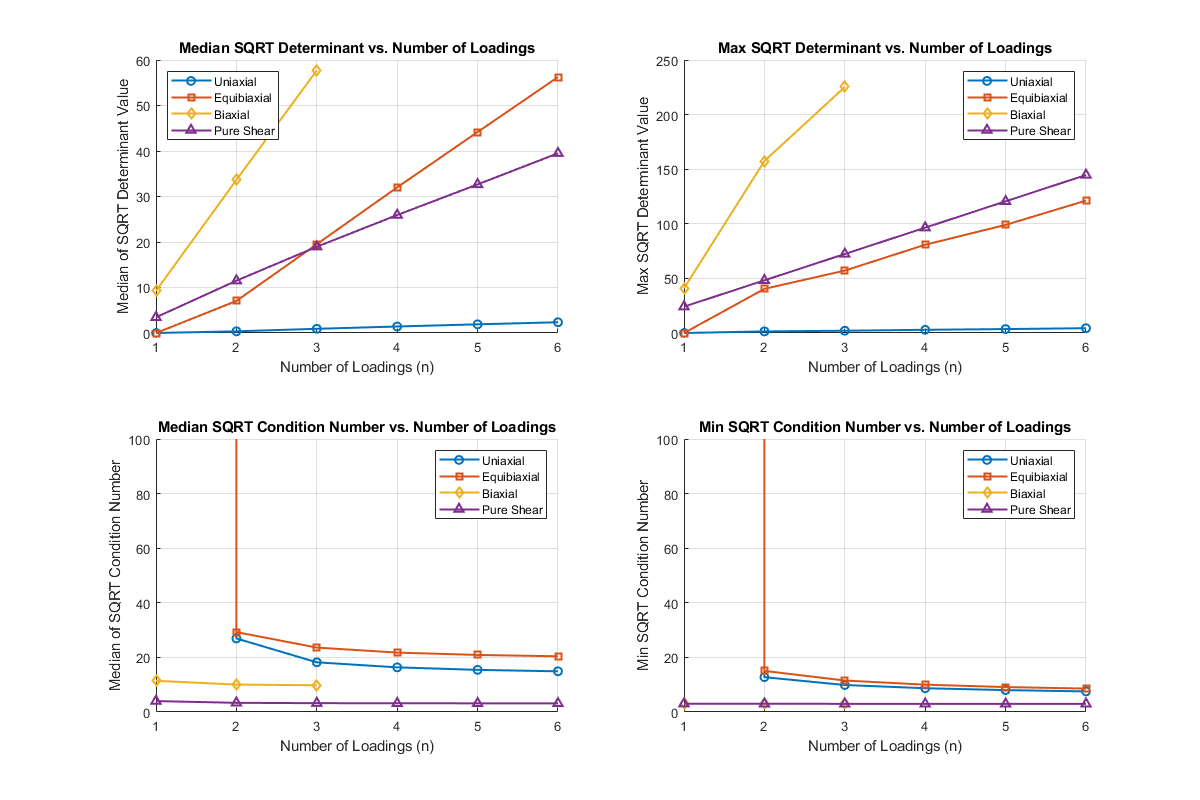


**Figure S8.** Max and median of SQRT determinant, and min and median SQRT condition number vs the number of measurements for Ogden model with $N=2, \alpha_{1}=3, \alpha_{2}=-3$ and and stretch ranges between 1 and 2 for different loading modes.


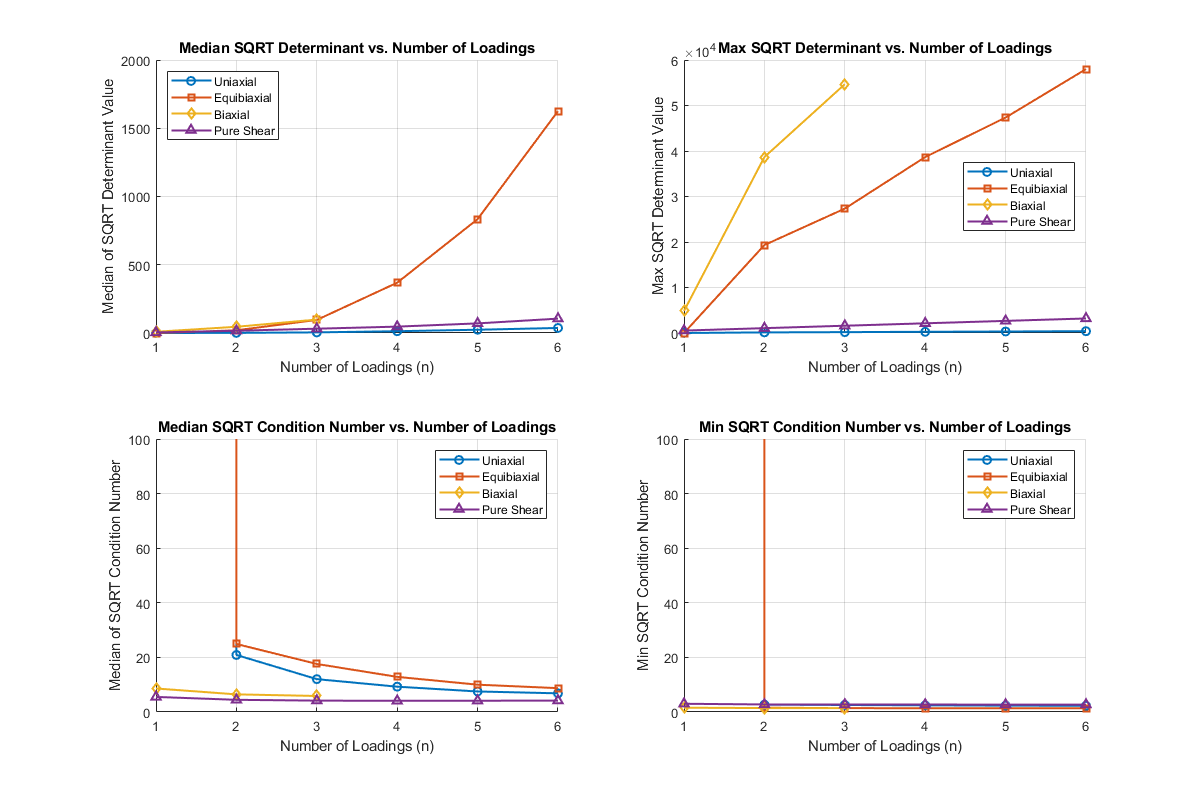


**Figure S9.** Max and median of SQRT determinant, and min and median SQRT condition number vs the number of measurements for Ogden model with $N=2, \alpha_{1}=3, \alpha_{2}=-3$ and and stretch ranges between 0.4 and 2 for different loading modes.


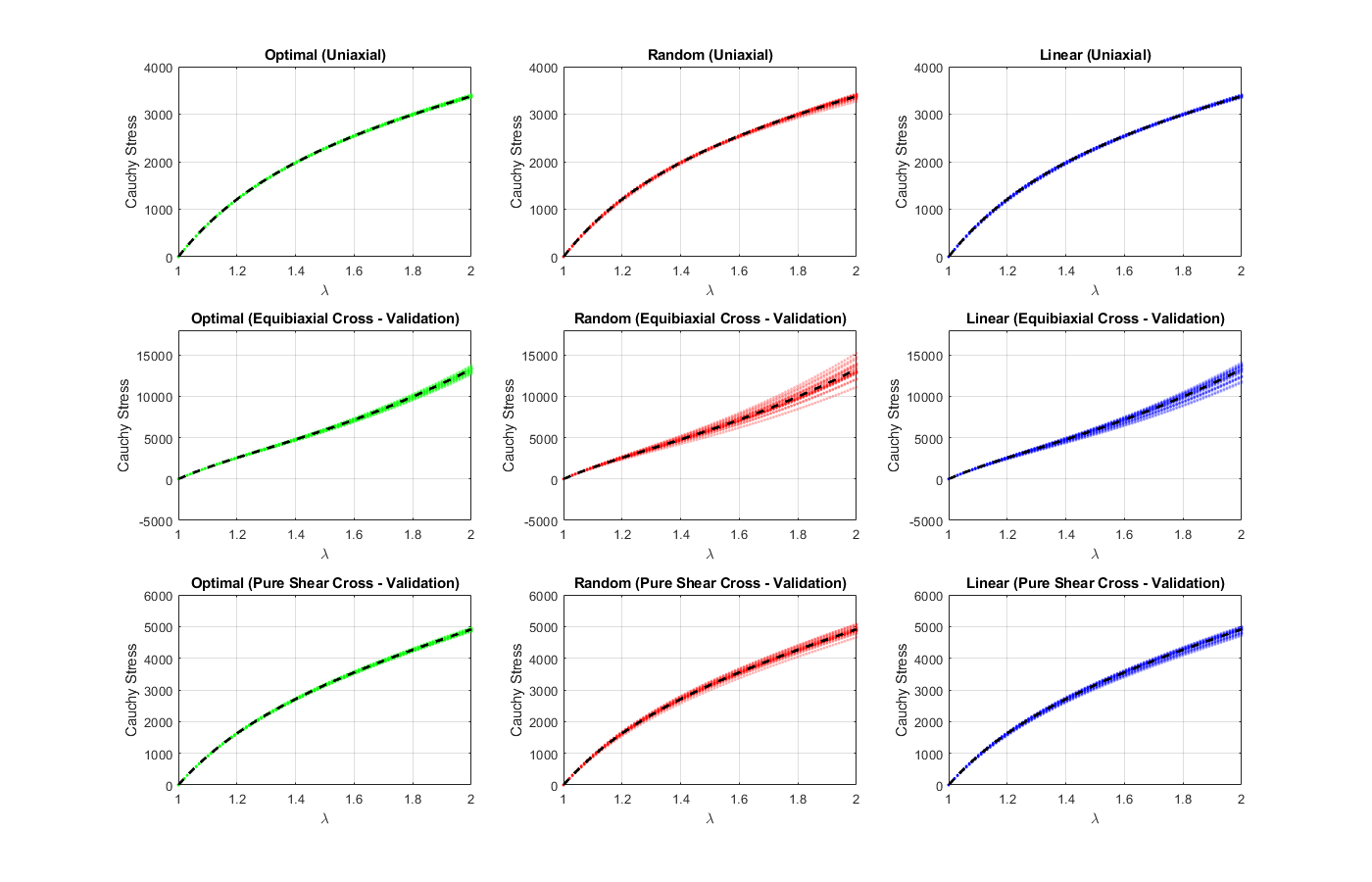


**Figure S10.** Results of repeating the uniaxial characterization with 3 measurements for 20 times on a) optimally selected stretches; b) randomly selected stretches; and c) linearly distributed stretches. the synthetic Mooney-Rivlin data with brain material parameters reported by Mendis et. al., is used as ground truth and 0.5% gaussian stress measurement error is added. The stretch range is 1 to 2.


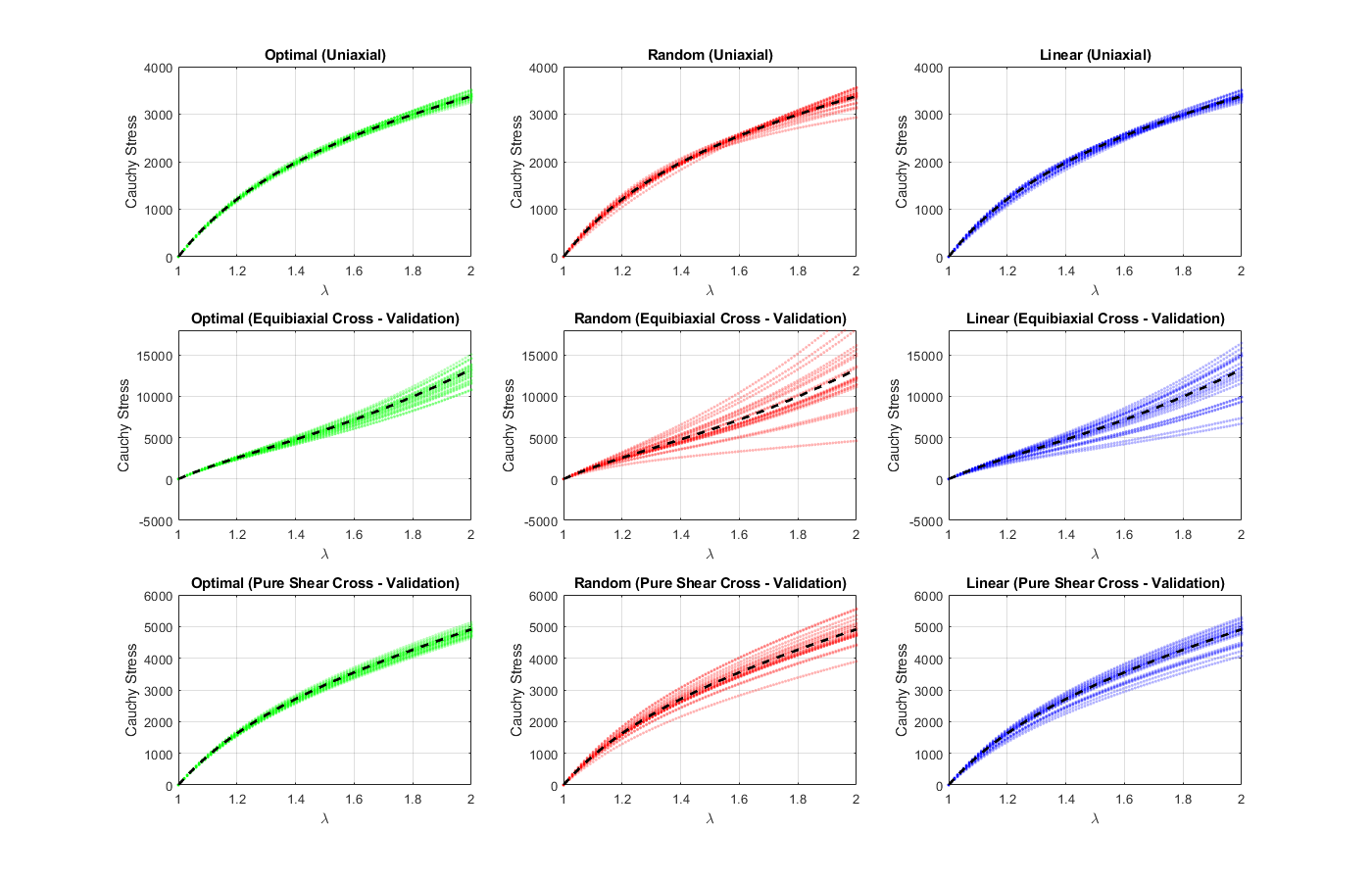


**Figure S11.** Results of repeating the uniaxial characterization with 3 measurements for 20 times on a) optimally selected stretches; b) randomly selected stretches; and c) linearly distributed stretches. the synthetic Mooney-Rivlin data with brain material parameters reported by Mendis et. al., is used as ground truth and 2% gaussian stress measurement error is added. The stretch range is 1 to 2.


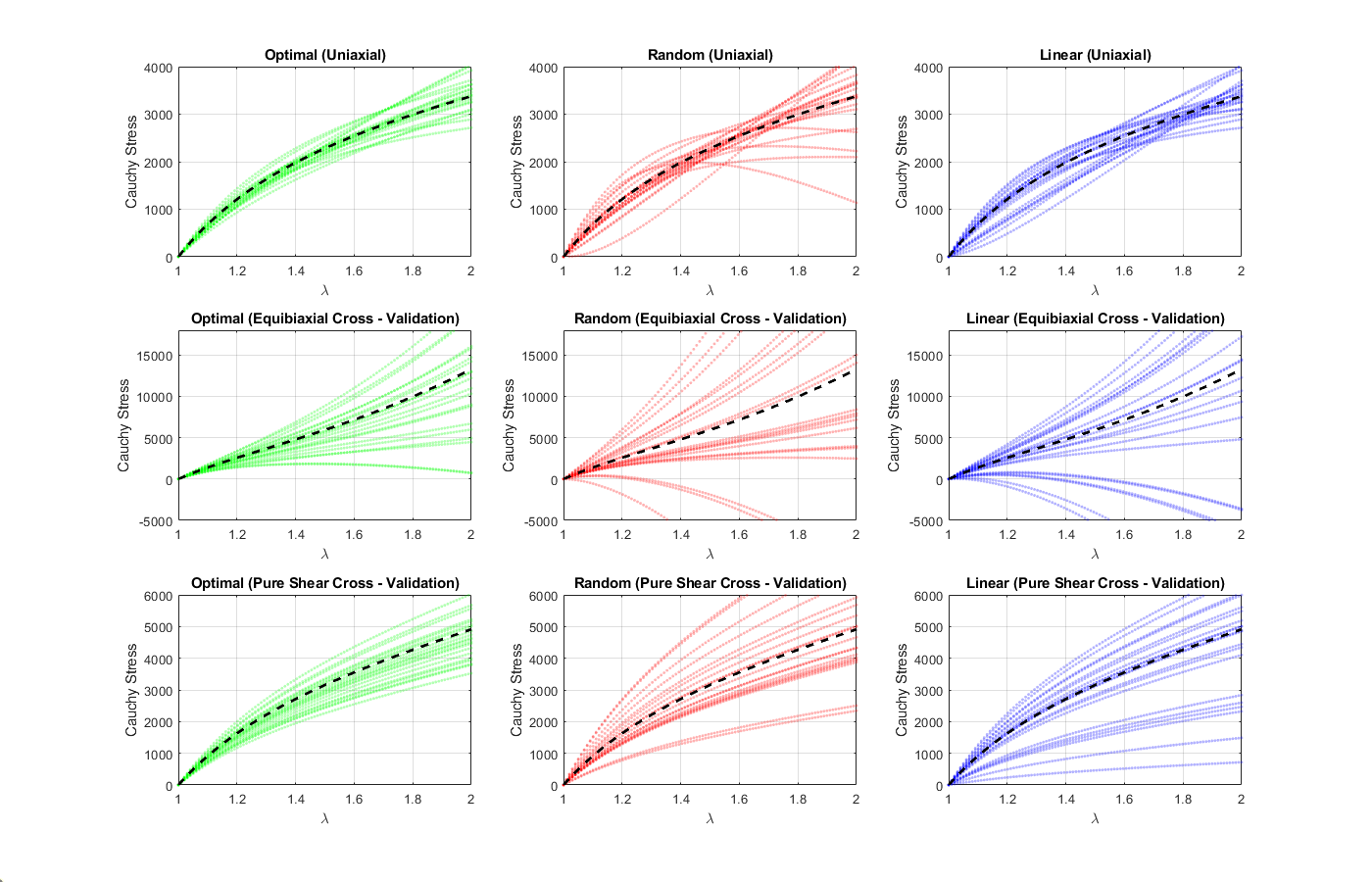


**Figure S12.** Results of repeating the uniaxial characterization with 3 measurements for 20 times on a) optimally selected stretches; b) randomly selected stretches; and c) linearly distributed stretches. the synthetic Mooney-Rivlin data with brain material parameters reported by Mendis et. al., is used as ground truth and 10% gaussian stress measurement error is added. The stretch range is 1 to 2.


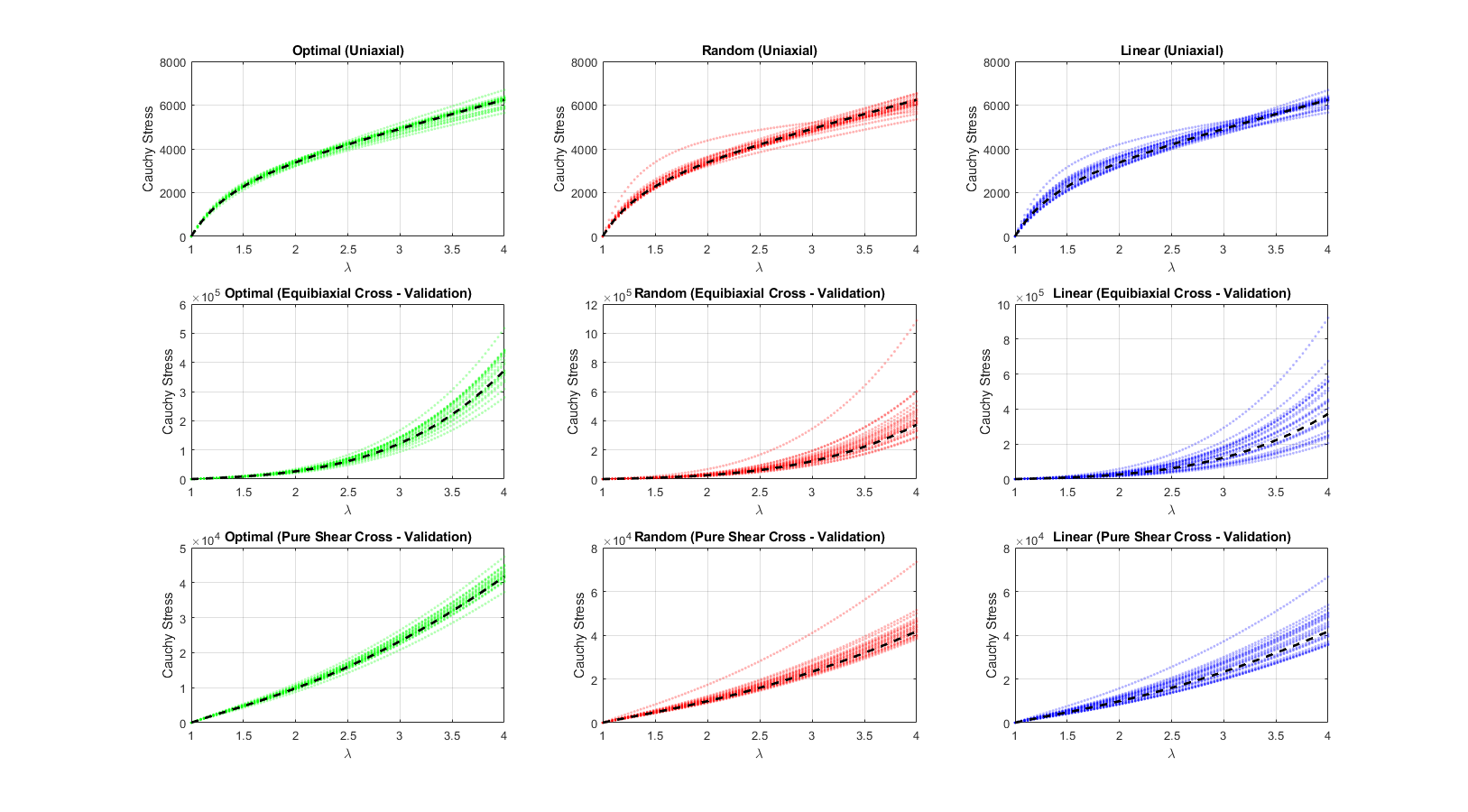


**Figure S13.** Results of repeating the uniaxial characterization with 3 measurements for 20 times on a) optimally selected stretches; b) randomly selected stretches; and c) linearly distributed stretches. the synthetic Mooney-Rivlin data with brain material parameters reported by Mendis et. al., is used as ground truth and 5% gaussian stress measurement error is added. The stretch range is 1 to 4.


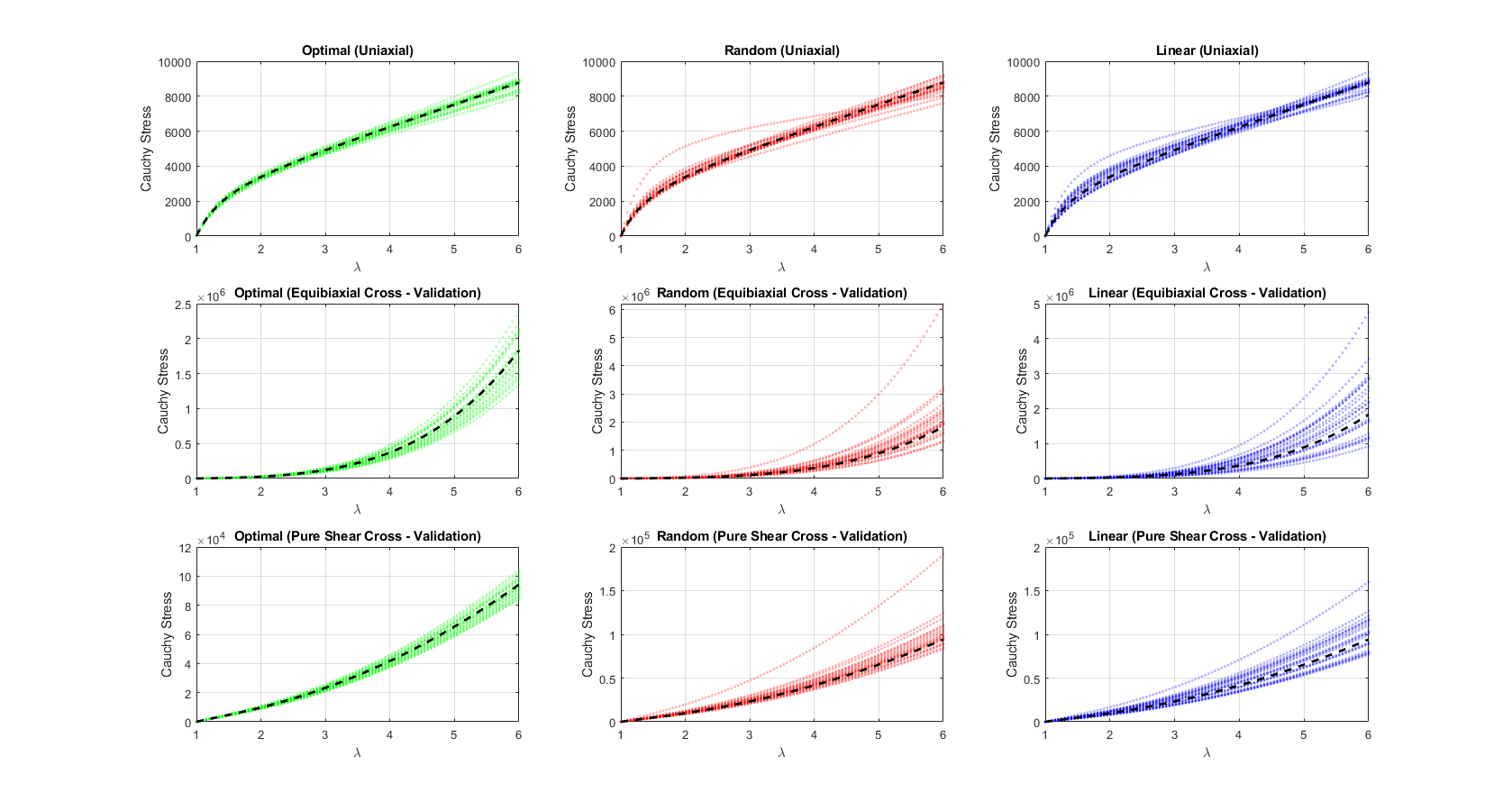


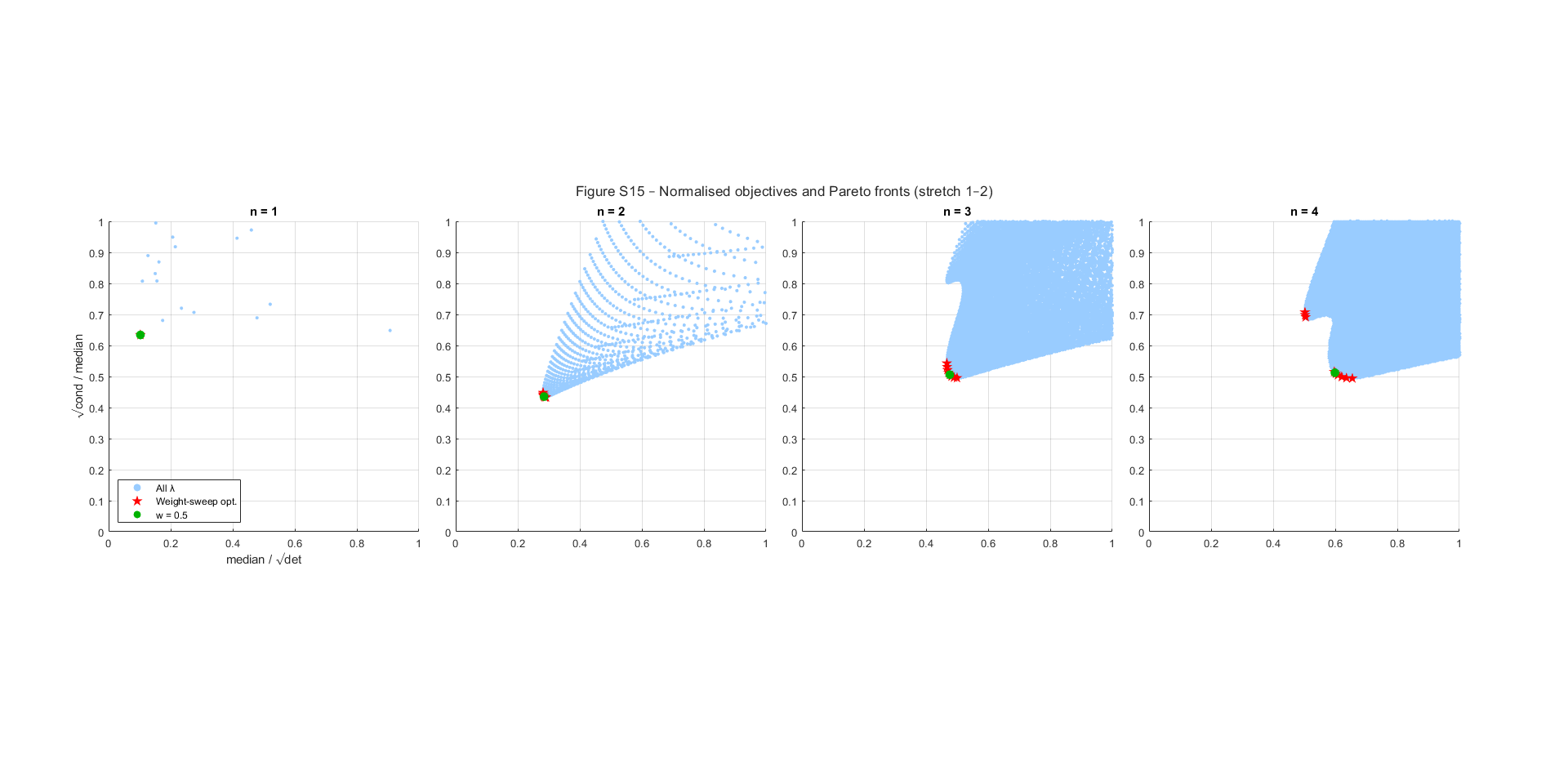
**Figure S14.** Results of repeating the uniaxial characterization with 3 measurements for 20 times on a) optimally selected stretches; b) randomly selected stretches; and c) linearly distributed stretches. the synthetic Mooney-Rivlin data with brain material parameters reported by Mendis et. al., is used as ground truth and 5% gaussian stress measurement error is added. The stretch range is 1 to 6.

**Figure S15.** The pareto front has been demonstrated on different number of measurements for a Mooney-Rivlin model under uniaxial tension with stretch range of 1 to 2. The red stars correspond to the weight values between 0 and 1 sweeping the front which satisfactorily capture the tip. The green dots correspond to weight value of 0.5 which supports the use of this weight for the optimizations within the manuscript.
